# Radiochemotherapy-induced DNA repair promotes the biogenesis of gastric cancer stem cells

**DOI:** 10.1186/s13287-022-03165-8

**Published:** 2022-09-24

**Authors:** Yu Lu, Xiaobo Zhang

**Affiliations:** College of Life Sciences, Laboratory for Marine Biology and Biotechnology of Pilot National Laboratory for Marine Science and Technology (Qingdao) and Southern Marine Science and Engineering Guangdong Laboratory (Zhuhai), Zhejiang University, Hangzhou, 310058 People’s Republic of China

**Keywords:** DNA repair, Gastric cancer stem cells, EID3, NAMPT, Wnt signaling pathway

## Abstract

**Background:**

Clinically, metastasis and recurrence occurred after routine radiochemotherapy in dozens of cases of gastric cancer, mainly attributed to the role of cancer stem cells (CSCs). Actually, radiochemotherapy could induce DNA damages, leading to activation of DNA repair which might be associated with acquisition of stem cell phenotype. Hitherto, the contribution made by active DNA repair to stemness induction has not been extensively explored.

**Methods:**

Cisplatin/doxorubicin treatment and X-ray exposure were conducted in gastric cancer cell lines and gastric cancer cells derived from solid tumors to model clinical therapy. Quantitative real-time PCR, Western blot, and tumorsphere/tumor formation assay were further used to characterize CSCs and assess activation of DNA repair. RNA-seq was performed to identify which DNA repair genes were crucial for CSC traits induction, followed by the investigation of underlying mechanism and functional significance via in vitro and in vivo experiments.

**Results:**

Here, we report a mechanism through which gastric cancer cells in response to radiochemotherapy were reprogrammed into gastric cancer stem cell-like cells. In this mechanism, radiochemotherapy triggers DNA damage response accompanied by elevated levels of EID3, a typical DNA repair gene, which interacts with NAMPT to promote stemness via upregulating Wnt signaling pathway, manifested by enhanced tumorsphere/tumor formation in gastric cancer. In addition to involvement of EID3 in stemness acquisition, it also shows impacts on proliferation, cell cycle, apoptosis and therapy resistance to maintain the characteristics of CSC populations.

**Conclusion:**

Our study indicates that gastric cancer cells can be endowed with stemness traits via EID3-NAMPT-Wnt/β-catenin axis in response to radiochemotherapy. Blocking this axis (*i.e.*, targeting EID3) along with radiochemotherapy might represent a potential strategy to sensitize CSCs to radiochemotherapy and further reinforce the anti-tumor effects of conventional treatments.

**Supplementary Information:**

The online version contains supplementary material available at 10.1186/s13287-022-03165-8.

## Background

Radiotherapy and chemotherapy have been broadly used in cancer treatment for years. It is to be regretted that a subgroup of cancer cells can acquire resistance to drug upon treatments, and furthermore, ionizing radiation can lead to the deterioration of biological behavior of cancer cells and increase the capacity of cancer cells to invade and metastasis [1]. Clinical case analysis indicates that there emerge more cases of postoperative metastasis when receiving radiotherapy before surgery or surgery combined with radiotherapy than surgery alone in nasopharyngeal carcinoma, laryngeal cancer and breast cancer [2, 3, 4]. In addition to the current dilemma of radiotherapy, more than 90% of patients with metastatic tumors are considered to experience failed chemotherapy due to multidrug resistance [5]. Actually, drug resistance in cancer can lead to an immediate restart of the disease or relapse after a considerable period of time [6]. In this regard, a large number of patients have been facing recurrence after platinum-based combination chemotherapy [7]. It is believed that chemotherapy and radiotherapy resistance may result from the development of cancer stem cells (CSCs), a rare subpopulation of cancer cells sharing multiple characteristics with normal stem cells, for instance, the ability to self-renew for maintaining an undifferentiated state [8, 9].

Previous work has established that CSCs may originate from malignant transformation of adult stem cells [10] or epithelial–mesenchymal transition (EMT) dedifferentiation of cancer cells [11]. Recently, it has been found that differentiated cells can be transformed into CSCs with carcinogenicity by endogenous reprogramming [12]. Hitherto, the origins of CSCs have caused widespread controversy. Although the origins of CSCs differ, CSCs derived from diverse cancers share several critical transcription factors, which act as regulatory switches of CSCs, such as POU3F2, SOX2, SALL2 and OLIG2 [13]. In breast cancer, inhibition of transcription factor KLF4 (Kruppel-like factor 4) can suppress the self-renewal of breast CSCs, suggesting that KLF4 plays a part in maintaining the characteristics of CSCs with the involvement of Notch signaling pathway [14]. Similarly, octamer-binding transcription factor 3/4 (OCT3/4) maintains the pluripotency of pancreatic cancer cells [15]. Beyond that, transcription factor Nanog is thought to be a stemness marker for ovarian cancer and liver cancer for its capability to modulate stemness [16, 17]. It is believed that expression of these transcription factors maintaining the stemness might be associated with poor prognosis and low survival rate [18]. Recently, transcription factor YB-1 has been considered as an oncogenic protein upregulated in melanoma stem cells and found to stimulate proliferation of cancer stem cell [19], suggesting its key role in CSCs. Moreover, transcription factor NME/NM23 nucleoside diphosphate kinase 2 (NME2) can regulate the expression of C-myc responsible for the stemness regulation in Burkitt’s lymphoma [20]. There is ample evidence supporting that transcription factors play essential roles in CSCs. However, the regulatory mechanism of transcription factor expression and their impacts on CSCs have not been characterized yet.

The previous study of our laboratory revealed that NME2 is required for the maintenance of gastric cancer stem-like cell stemness [21]. To explore the regulatory mechanism for transcription factor expression in CSCs, the proteins bound to the promoter of *NME2* gene were characterized in gastric cancer stem cell-like cells in this study. The results revealed that XRCC5, XRCC6 and PARP1, essential proteins required for DNA repair [22], were interacted with the promoter of *NME2*, hinting the involvement of DNA repair in the biogenesis of CSCs. Thus, the relationship between DNA repair and CSCs was further investigated and the results demonstrated that DNA repair, induced by chemotherapy and radiotherapy, could promote the biogenesis of CSCs.

## Methods

### Sorting of gastric cancer stem cell-like cells and non-stem cells from cell line

Gastric cancer cell line MKN-45 was used for the sorting of gastric cancer stem cell-like cells and the corresponding non-stem cells using ALDEFLUOR kit (STEMCELL Technologies, Canada) via fluorescence-activated cell sorting (FACS). In brief, MKN-45 cells purchased from American Type Culture Collection (ATCC) were collected, washed and incubated in ALDEFLUOR buffer containing an ALDH1 (aldehyde dehydrogenase 1) substrate-BODIPY-aminoacetate (BAAA) with or without the addition of diethylaminobenzaldehyde (DEAB), a specific ALDH inhibitor. FACS was then conducted on an Aria cell sorter (BD Biosciences, USA) at an excitation of 575 nm. The ALDH1-positive cells were sorted for later use.

### Cell culture

Gastric cancer non-stem cells (GCNSCs) were cultured in Leibovitz’s L-15 medium and Dulbecco’s modified eagle medium (DMEM) (Sigma-Aldrich, USA) supplemented with 10% fetal bovine serum (FBS). Gastric cancer stem cell-like cells (GCSCs) were cultured in Dulbecco’s modified eagle medium/nutrient mixture F12 (DMEM/F12) (Invitrogen, USA) supplemented with 20 ng/mL epidermal growth factor (EGF) (Beyotime Biotechnology, Shanghai, China), 10 ng/mL basic fibroblast growth factor (bFGF) (Beyotime Biotechnology), 5 μg/mL of insulin (Beyotime Biotechnology), and 2% of B27 (Sigma-Aldrich). Gastric cancer non-stem/stem cells grew at 37 °C in a humidified atmosphere with 5% CO_2_.

### Isolation of nuclear proteins

GCSCs (1 × 10^7^) were treated with 3 mL trypsin–ethylenediaminetetraacetic acid (trypsin–EDTA) (Gibco, USA) for 2 min, respectively. Then, the cells were centrifuged at 1000×*g* for 5 min, followed by washes with phosphate-buffered saline (PBS). After completely aspirating PBS, 800 μL HEPES (4-(2-hydroxyethyl)-1-piperazineethanesulfonic acid) buffer (10 mM HEPES, 1.5 mM MgCl_2_, 10 mM KCl, pH 7.9) was added to each sample and placed in ice for 2 min, followed by the addition of nonidet P-40 (NP-40) (Sigma-Aldrich, USA) at 0.4% final concentration. Samples were centrifuged at 1000×*g* for 7 min and then resuspended in 500 μL HEPES buffer. This step was repeated three times. Subsequently, the supernatant was mixed with buffer C (1 M HEPES, 1 M MgCl_2_, 5 M NaCl, 0.5 M EDTA, 1 M dithiothreitol, 250 mL glycerol, 3 μL phenylmethanesulfonyl fluoride, pH8.0). The sample was incubated at 4 °C for 4–6 h. After centrifugation at 5000×*g* for 5 min, the supernatant containing nuclear proteins was collected for later use.

### Streptavidin-agarose pulldown assay and protein identification

The synthesized biotin-labeled double-stranded DNA of NME2 promoter was mixed with the extracted nuclear proteins and streptavidin-agarose beads (Sigma-Aldrich, USA) at 4 °C for 4–6 h. As a negative control, an intragenic double-stranded DNA amplified with sequence-specific primers (5′-CACCTTCATCGCCATCAAGC-3′ and 5′-GACCCAGTCATGAGCACAAGA-3′) was coupled to Streptavidin-agarose beads and included in the assays. Subsequently, the agarose beads were centrifuged at 600×*g* for 1 min and washed with phosphate-buffered saline (PBS). The supernatant was subjected to SDS-PAGE with Coomassie staining. The proteins were identified by mass spectrometry.

### Western blot

Proteins were separated by 10% SDS-PAGE and then transferred to a polyvinylidene difluoride (PVDF) membrane. After blocking with 5% skim milk in Tris-buffered saline (137 mM NaCl, 20 mM Tris–HCl, pH8.0) containing 0.05% Tween-20 (Sangon Biotech, Shanghai, China), the membrane was incubated with a primary antibody (Abcam, Cambridge, UK) overnight at 4 °C. Subsequently, the membrane was incubated with HRP (horseradish peroxidase)-conjugated secondary antibody (Abcam) at 37 °C for 2 h, followed by examination with enhanced chemiluminescence system (ChampGel, Beijing, China).

### Cisplatin, doxorubicin, X-ray or actinomycin D treatment

Gastric cancer non-stem cells (GCNSCs) were seeded onto a 6-well plate at 2 × 10^5^ cells/well in triplicate and cultured in DMEM medium containing 10% FBS overnight. Subsequently the cells were treated with chemotherapeutics (300 μM cisplatin or 0.5 μM doxorubicin) (Sigma-Aldrich, USA) or exposed to 20 Gy X-ray (Rad Source Technologies, USA). Twenty-four hours later, the cells were collected to conduct tumorsphere formation assay and the detection of gene expression using quantitative real-time PCR and Western blot. To assess the cellular characterizations of cancer stem cell-like cells, the radiotherapy- or chemotherapy-treated GCNSCs were cultured in serum-free medium. After culture for 7 days, the individual cells forming larger tumorspheres and highly expressing stemness genes were collected and considered as GCSCs (cisplatin-induced GCSCs, doxorubicin-induced GCSCs or X-ray-induced GCSCs). The radiotherapy- or chemotherapy-induced GCSCs were used for tumorigenicity in nude mice, cell proliferation, cell cycle, apoptosis and therapy resistance assays.

For the actinomycin D treatment, GCNSCs (2 × 10^5^ cells/well) were simultaneously treated with cisplatin, doxorubicin or X-ray and DNA repair inhibitor actinomycin D (5 μM) and then incubated for 24 h. GCNSCs without the actinomycin D treatment were used as controls. The cells were harvested for Western blot analysis.

### Quantitative real-time PCR

Total RNAs were extracted from cells using RNA Isolation Kit (Ambion, USA). Reverse transcription was conducted with PrimeScript RT Reagent Kit (Takara, Japan). Subsequently, quantitative real-time PCR was performed using sequence-specific primers [ERCC4 (excision repair 4, endonuclease catalytic subunit), 5′-TCCTCAGTTGAACCTCCGTAT-3′ and 5′-ACCCCTCACTATCATCCATCC-3′; XPD (xeroderma pigmentosum D), 5′-AGACTGGAAACGCTCAAATAC-3′ and 5′-TGCCACCCTTGTCAGCACCTA-3′; BRCA1 (breast cancer susceptibility 1), 5′-TGTGAGAGAAAAGAATGGAATAAGC-3′ and 5′-CATCATGTGAGTCATC AGAACCTAA-3′; BRCA2 (breast cancer susceptibility 2), 5′-TATTT GGTGCCAC AACTCCTT-3′ and 5′-TTACCTCCACCTGTTAGTCCC-3′; PARP1 (poly ADP-ribose polymerase 1), 5′-TCGCCTTCACGCTCTATCTTA-3′ and 5′-TGGAA ATGCTTGACAACCTGC-3′; RAD51 (RAS associated with diabetes protein 51), 5′-ATTTTGCAGATTCTGGTTTCC-3′ and 5′-TCGCTGATGAGTTTGGTGTAG-3′; ALDH1, 5′-CATTGTCCAAGTCGG CATCAG-3′ and 5′-GGCAGCCATTTCTTCTCACAT-3′; OCT-3/4, 5′-TCTTTCTGTCCTTTCACGATGCTCT-3′ and 5′-TCATTCACCC ATTCCCTGTTCACT-3′; SOX2 (SRY-box 2), 5′-AAAATCCCATCACCCACAGC AA-3′ and 5′-AAAATAGTCCCCCAAAAAGAAGTCC-3′; GAPDH (glyceraldehyde-3-phosphate dehydrogenase), 5′-GAAGGTGAAGGTCGGAGTC AAC-3′ and 5′-CAGAGTTAAAAGCAGCCC TGGT-3′] and TagMan Universal PCR Master Mix (Takara, Japan). GAPDH was used for normalization. PCR was conducted by maintaining at 95 °C for 30 s, followed by alternating for 40 thermal cycles between 95 °C for 5 s and 60 °C for 30 s. The relative fold changes of mRNA expression were calculated based on $2^{-(\Delta\Delta {{\text{C}}_{{\text{t}}}})}$ method [23].

### Tumorsphere formation assay

Tumorsphere formation assay was conducted under non-adherent and serum-free conditions. GCSCs were seeded in a 6-well plate at 10^3^ cells/mL and cultured in DMEM/F12 medium (Gibco, USA) supplemented with 20 ng/mL EGF (Sigma, USA), 10 ng/mL bFGF (Sigma), 5 μg/mL of insulin (Sigma) and 2% of B27 (Sigma). After 5 days of culture, the forming tumorspheres were scattered in DMEM/F12 medium. A single cell from a tumorsphere was plated into a 96-well plate and cultured for 2 weeks. The tumorsphere was examined under a light microscope. A total of 200 single cells were assayed to evaluate the percentage of tumorsphere formation.

### Isolation of cancer stem cells and non-stem cells from solid tumors of patients with gastric cancer

The solid tumors of two patients with gastric cancer were rinsed with phosphate-buffered saline (PBS) (Invitrogen, USA). Subsequently, the tumors were cut into 1 mm^3^ mincemeat, followed by digestion for about 5 h using collagenase I (Gibco, USA). The mixture was filtrated with a 200-mesh filter, and the filtrate was centrifuged at 100×*g* for 10 min. The supernatant, the single cell suspension, was cultured in serum-free DMEM/F12 medium (Gibco, USA), a medium widely used for the growth of cancer stem cells, containing 2% of B27 (Invitrogen, USA), 5 μg/ml insulin (Sigma, USA), 20 ng/ml bFGF (Sigma, USA), 20 ng/ml EGF (Sigma, USA) and 1% penicillin–streptomycin solution (Gibco, USA). Seven days later, the cells collected from tumorspheres were considered as gastric cancer stem cell-like cells (GCSCs) and further cultured in serum-free medium. The isolated GCSCs were further confirmed by the examination of stemness genes’ expressions using Western blots. The cells, which did not form tumorspheres, were collected as gastric cancer non-stem cells and cultured in DMEM basic medium (Gibco, USA) with 10% fetal bovine serum (FBS) (Gibco, USA) and 1% penicillin–streptomycin solution (Gibco, USA).

### Online data mining

To characterize the gene expression in the cancerous tissues of cancer patients and healthy donors, the Cancer Genome Atlas (TCGA) database (http://cancergenome.nih.gov) was used with UALCAN (http://ualcan.path.uab.edu/index.html).

The correlation of the EID3 expression level with the overall survival of gastric cancer patients was analyzed by the Kaplan–Meier Plotter (http://www.kmplot.com/). The gastric cancer patients (ID: 231292_at) were divided into EID3-low group (*n* = 282) and EID3-high group (*n* = 349). The hazard ratio (HR) with 95% confidence intervals and logrank p value were also computed.

### RNA-seq analysis

Total RNAs were extracted from gastric cancer non-stem cells (GCNSCs), cisplatin-induced gastric cancer stem cell-like cells (GCSCs), doxorubicin-induced GCSCs, X-ray-induced GCSCs and the sorted GCSCs using TRIzol reagent (Invitrogen, Carlsbad, USA) according to the manufacturer’s instruction. Then the extracted RNAs were treated with DNase I (Invitrogen) to remove DNA and subjected to sequencing by Majorbio Biomedical Science and Technology Co., Ltd. (Shanghai, China). Data processing of raw reads was quality-checked by utilizing fastqc v0.10.1 (http://www.bioinformatics. babraham.ac.uk/projects/fastqc/) and trimmed for low-quality bases and adaptors if necessary using fastx toolkit V0.0.13 (http://hannonlab.cshl.edu/fastx_toolkit/). The raw data were aligned using TopHat v2.0.4 with default options and then assembled using Cufflinks v2.2.1.

The differential expression analysis of genes was performed with the DESeq2 package (v1.20.0). The differentially expressed genes were assigned based on fold change > 2 and *P* adjust < 0.05. Following the identification of the significantly differentially expressed genes, the volcano plots and heat map were constructed on the normalized gene expression data to visualize the certain genes expression intensity data.

### Correlation analysis

Spearman correlation coefficient and p value were calculated using corrplot package in R [24]. When p value was smaller than 0.05, the difference between treatments was considered to be high confidence. A correlation coefficient greater than 0 was regarded as a positive correlation.

### Coimmunoprecipitation (Co-IP) assay

Cells were lysed in ice-cold lysis buffer (Beyotime Biotechnology, Shanghai, China) containing 2 mM protease inhibitor phenylmethylsulfonyl fluoride (PMSF) (Solarbio Life Sciences, Beijing, China). The lysate was incubated with EID3-specific antibody (Abcam, Cambridge, UK) at 4 °C overnight. Then, protein G-agarose beads (Invitrogen, Carlsbad, USA) were added into the mixture, followed by incubation at 4 °C for 4 h. After washes with ice-cold lysis buffer, the Co-IP product was subjected to SDS-PAGE with Coomassie brilliant blue staining. The distinct protein bands were identified using mass spectrometry analysis.

### Cell proliferation assay

Cell proliferation assays were conducted using cell counting kit-8 (CCK-8) (Dojindo Laboratories, Tokyo, Japan). The cells (1 × 10^4^ cells/mL) were added with CCK-8 solution (Dojindo) and then incubated at 37 °C in a humidified incubator containing 5% CO_2_ for 2 h. The absorbance of cells was examined at 450 nm.

### Silencing and rescue of gene expression in cells

To knock down the expression of aprataxin and PNKP-like factor (APLF), EP300 interacting inhibitor of differentiation 3 (EID3), essential meiotic structure-specific endonuclease subunit 2 (EME2), neuronal PAS domain protein 2 (NPAS2) or DNA polymerase nu (POLN) in cells, RNAi assay was performed using the gene-specific siRNA (APLF-siRNA, 5′-AUAGAUUUGGCUUCAAUGGUA-3′; EID3-siRNA, 5′-AAUCAGAAGCCAUAACAAGAA-3′; EME2-siRNA, 5′-CAGGAUCAGUGUCU CUCAAGC-3′; NPAS2 siRNA, 5′-AUUUUCUAUGCAGUUUUUCCU-3′; POLN-siRNA, 5′-UUCCAUUUUCACAAAAUCCUC-3′). Cells (2 × 10^5^) were transfected with 100 nM siRNA using Lipofectamine 2000 (Invitrogen, Carlsbad, USA). As a control, the siRNA control (5′-UUCUCCGAACGUGUCACGUTT-3′) was included in the transfection. At different times after transfection, the cells were collected.

To rescue the expression of EID3 in EID3-silenced cells, the *EID3* gene was cloned into pcDNA3.1( +) plasmid (Invitrogen, USA) using sequence-specific primers (5′-ATGAAGATGGATGTGTCAGTGA-3′ and 5′-TTCCCCAGAGCT GAGGTCGT CG-3′); 5′-GCCGACGTAGACCCAAAGCTCCT-3′ and 5′-TTAGTATGAGGAG TA TGTAAT-3′). The *EID3* gene was mutated at position 256 from G to A to avoid the recognition by EID3-siRNA. Subsequently, the recombinant plasmid was transfected into the EID3-knocked-down cells with Lipofectamine 2000 (Invitrogen). At different time after transfection, the cells were collected for later use.

### KEGG (Kyoto Encyclopedia of Genes and Genomes) analysis

To reveal the pathways involved by a protein, KEGG analysis was performed on the module of “KEGG PATHWAY” (https://www.kegg.jp/). Then, the signaling pathways were obtained.

### Cell cycle analysis

Cell cycle analysis was performed using flow cytometry. Cells were fixed in 70% ethanol (ice-cold) for at least 1 h, and then the cells were incubated with DNase-free RNase A (20 μg/ml) (BD Biosciences, San Jose, USA) for 30 min at room temperature. After centrifugation at 500×*g* for 5 min, the pellet was stained with propidium iodide (PI) (50 μg/ml) (Sigma, St. Louis, USA). The fluorescence intensity of 1 × 10^4^ cells was measured with a flow cytometer.

### Caspase 3/7 activity analysis

Caspase-Glo 3/7 was used to examine the activity of caspase 3/7 of cells according to the manufacturer’s recommendations (Promega, USA). Cells (1 × 10^3^/well) were plated into a 96-well plate and cultured for 36 h, followed by the addition of 100 μl Caspase-Glo 3/7 reagent (Promega, USA). After incubation in the dark at room temperature for 1 h, the luminescence of cells was measured using a microplate reader (Promega, USA).

### Apoptosis detection with annexin V

Cells were harvested and washed with cold PBS. Subsequently, the cells were stained with fluorescein isothiocyanate (FITC)-annexin V and propidium iodide (PI) using a FITC Annexin V apoptosis detection kit (BD Biosciences, USA) according to the manufacturer’s protocol. After incubation at room temperature for 15 min in the dark, the sample was immediately analyzed using flow cytometry at an excitation of 575 nm with calculation of percentage of apoptotic cells.

### Immunofluorescence analysis

Cells were fixed in 4% paraformaldehyde for 30 min, permeabilized with 0.3% Triton X-100 for 10 min and blocked with 5% bovine serum albumin (BSA) for 2 h. Subsequently, the cells were stained overnight at 4 °C with a primary antibody (diluted at 1:200 in 5% BSA) (Abcam, UK), followed by incubation with the fluorophore-conjugated secondary antibody (Abcam) for 2 h at room temperature. The nuclei were stained with 4′,6-diamidino-2-phenylindole (DAPI) for 10 min. The cells were examined and imaged using a laser scanning confocal microscope (Olympus GmbH, Germany).

### Tumorigenicity in nude mice

GCSCs were transfected with shRNA control, EID3-shRNA or EID3-shRNA + plasmid expressing EID3. Two weeks later, the cells were resuspended in physiological saline and then matrigel (Becton, Dickinson and Company, USA) was added to the cell suspension a ratio of 1:2. About 100–150 μL of the cell suspension was subcutaneously injected into BALB/c mice. The tumor volume in mice was measured every week. Seven weeks later, the mice were killed and the tumor sizes and weights were examined. Animal experiments in this study were approved by The Animal Experiment Center of Zhejiang University, China. All methods were carried out in accordance with approved guidelines.

### Statistical analysis

All numerical data were presented as mean ± standard deviations (SD). One-way analysis of variance (ANOVA) and Student’s t test were employed to determine the statistical significance of differences between treatments. All assays were performed in biological triplicate.

## Results

### DNA repair is associated with the biogenesis of cancer stem cells

**Fig. 1:**
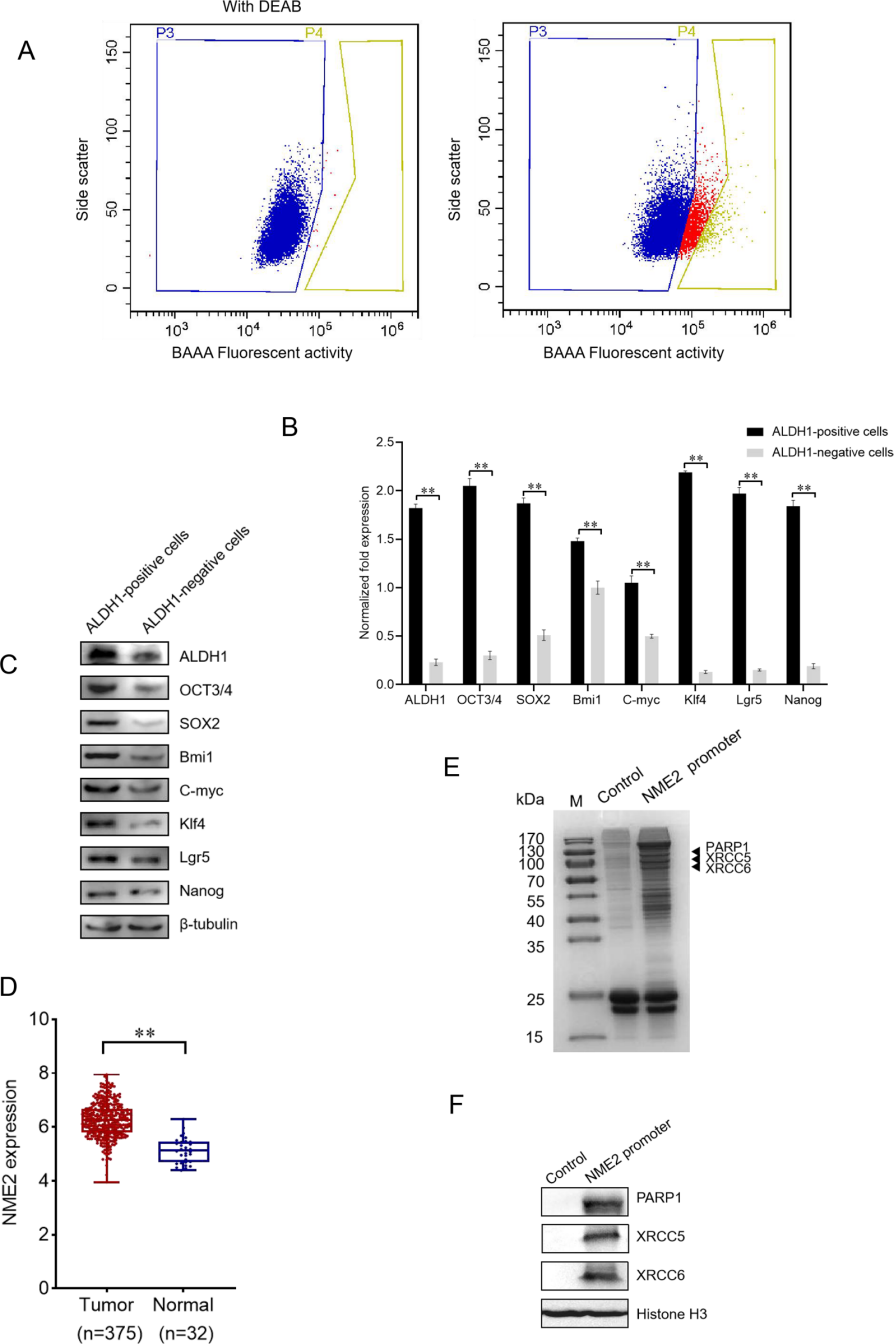
DNA repair is associated with the biogenesis of cancer stem cells. **A** Sorting of gastric cancer stem cell-like cells (GCSCs) from MKN-45 cell line. The fluorescence-activated cell sorting was performed based on the detection of ALDH1 activity using the ALDH1 fluorescent substrate BODIPY-aminoacate (BAAA). The ALDH1-positive cells were gastric cancer stem cell-like cells (P4 region) and ALDH1-negative cells were gastric cancer non-stem cells (P3 region). **B** Expression of stemness genes in ALDH1-positive cells. The gene expressions were examined with quantitative real-time PCR (**p < 0.01). **C** Expressions of stemness genes in ALDH1-positive cells and ALDH1-negative cells using Western blot. β-tubulin was used as a control. **D** Upregulation of NME2 in the cancerous tissues of gastric cancer patients. The gene expression of NME2 in the cancerous tissues of gastric cancer patients and healthy donors was analyzed with the Cancer Genome Atlas (TCGA) database. **E** Identification of proteins interacted with the promoter of transcription factor *NME2* gene. Nuclear proteins extracted from gastric cancer stem cell-like cells (MKN-45) were incubated with biotin-labeled dsDNA of NME2 promoter and streptavidin-agarose beads. An intragenic double-stranded DNA coupled to Streptavidin-agarose beads alone was used as a control. The proteins bound to NME2 promoter were separated by SDS-PAGE with Coomassie staining and then identified by mass spectrometry. Arrows indicated the identified proteins interacted with the NME2 promoter. M, protein marker. **F** Western blot analysis of the proteins bound to the promoter of NME2. The proteins of the pulldown eluates using the promoter of NME2 were subjected to Western blot analysis. Streptavidin-agarose beads coupled with an intragenic double-stranded DNA served as a control

To reveal the regulatory mechanism of transcription factor’s expression in cancer stem cells, gastric cancer stem cells were sorted from gastric cancer cell line MKN-45 using the marker ALDH1. The ALDH1-positive cells (Fig. 1A, P4 region) were identified as gastric cancer stem cell-like cells (GCSCs), while the ALDH1-negative cells (Fig. 1A, P3 region) were harvested as the corresponding non-stem cells. To confirm GCSCs, the expression of stemness genes was examined. The results revealed that the stemness genes were significantly upregulated in ALDH1-positive cells compared with the ALDH1-negative cells (Fig. 1B, C). These data indicated that GCSCs were obtained.

Based on the gene expression profile of NME2 in the cancerous tissues of cancer patients and healthy donors using the Cancer Genome Atlas (TCGA) database, NME2 was significantly upregulated in the cancerous tissues of gastric cancer patients compared with that of the healthy donors (Fig. 1D). These results demonstrated that NME2 played an important role in cancers.

To obtain the transcription factors of NME2, the promoter of *NME2* gene was used to screen the binding proteins in GCSCs. The results of promoter pulldown assays using the isolated nucleoproteins of GCSCs indicated that three proteins were specifically bound with the promoter of *NME2* (Fig. 1E). Based on mass spectrometry, the proteins bound to the promoter of *NME2* were identified to be XRCC5 (X-ray repair cross complementing 5), XRCC6 (X-ray repair cross complementing 6) and PARP1 (poly (ADP-ribose) polymerase 1) (Fig. 1E).

To confirm the mass spectrometry data, Western blot was conducted. The results revealed that the nuclear proteins interacted with the promoter of *NME2* were XRCC5, XRCC6 and PARP1 (Fig. 1F). As reported, XRCC5, XRCC6 and PARP1 are required for DNA repair [22], and PARP1 can act as a DNA-binding transcription factors [25]. In this context, the analysis of the transcription factor of *NME2* gene suggested that DNA repair might be associated with the biogenesis of cancer stem cells.

Collectively, these findings demonstrated that DNA repair was associated with tumorigenesis in clinic.

### Radiotherapy and chemotherapy promote DNA repair and biogenesis of cancer stem cells

**Fig. 2:**
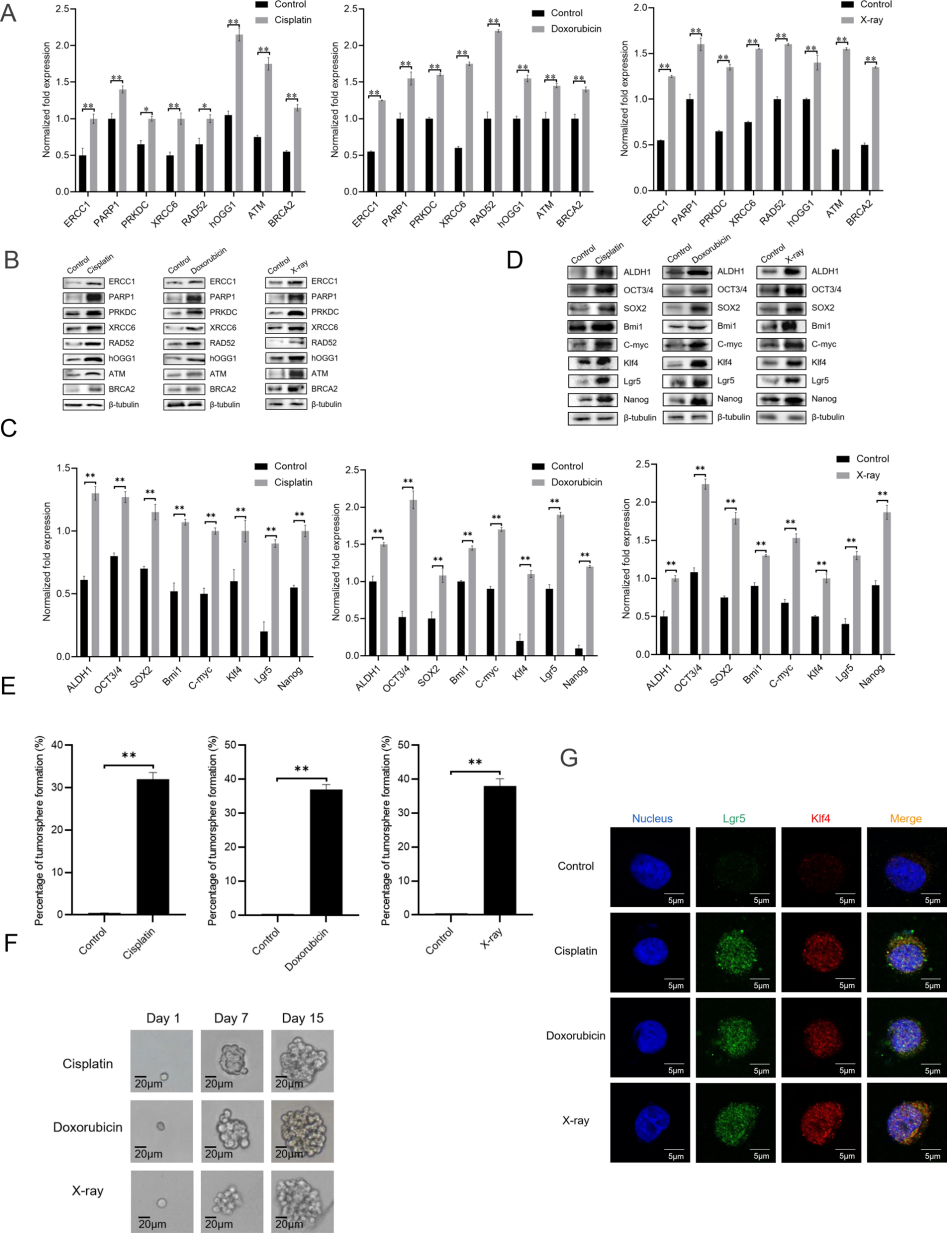
Radiotherapy and chemotherapy promote DNA repair and biogenesis of cancer stem cells. **A** Expressions of DNA repair genes in radiotherapy- or chemotherapy-treated gastric cancer non-stem cells (MKN-45, GCNSCs). The cells without any treatment were used as controls. The gene expression levels were examined with quantitative real-time PCR. **B** Western blot analysis of gene expression. β-tubulin was used as a control. **C** Expression profiles of stemness genes in radiotherapy- or chemotherapy-treated GCNSCs. The expressions of stemness genes in cisplatin-, doxorubicin- or X-ray-treated GCNSCs were determined by quantitative real-time PCR. The cells without any treatment were used as controls. **D** Upregulation of stemness genes in radiotherapy- or chemotherapy-treated GCNSCs. The cisplatin-, doxorubicin- or X-ray-treated GCNSCs were subjected to Western blot. β-tubulin was used as a control. **E** Percentage of tumorsphere formation of radiotherapy- or chemotherapy-treated GCNSCs. GCNSCs were treated with cisplatin, doxorubicin or X-ray. After culture for 7 days, the tumorsphere formation efficiency of radiotherapy- or chemotherapy-treated GCNSCs was evaluated. The data were the average values of three independent experiments. **F** Tumorsphere formation of a single tumor cell. A single cell from a tumorsphere of radiotherapy- or chemotherapy-treated GCNSCs was subjected to tumorsphere formation assay. At different time after culture, the tumorsphere was examined. Scale bar, 20 μm. In all panels, the significant differences between treatments were indicated with asterisks (*p < 0.05; **p < 0.01). **G** Immunofluorescence analysis of the radiotherapy- or chemotherapy-treated GCNSCs. GCNSCs (sorted from MKN-45) were treated with cisplatin, doxorubicin or X-ray for 48 h. As a control, GCNSCs alone were included in this assay. Subsequently, the cells were subjected to immunofluorescence staining to examine the stemness markers (Lgr5 and Klf4). Nuclei were stained with 4,6-diamidino-2-phenylindole (DAPI). Representative images of immunofluorescence staining were presented. Scale bar, 5 μm

To explore the relationship between DNA repair and the origin of CSCs, gastric cancer non-stem cells (GCNSCs) were treated with X-ray or chemotherapy drug (cisplatin or doxorubicin), followed by the detections of DNA repair and stemness gene expressions. The cells which could not form tumorspheres in the tumorsphere formation assay were collected and considered as GCNSCs. As shown in Fig. 2A, significant increases in mRNA levels of DNA repair genes were observed in cisplatin-, doxorubicin- or X-ray-treated GCNSCs, indicating that radiotherapy and chemotherapy caused DNA damage of GCNSCs to trigger the upregulation of DNA repair genes. Western blots yielded the similar results (Fig. 2B). At the same time, the stemness genes were significantly upregulated in cisplatin-, doxorubicin- or X-ray-treated GCNSCs (Fig. 2C, D). These data suggested that radiotherapy and chemotherapy in clinical experience could induce activated DNA repair, thus leading to the reprogramming of tumor cells to tumor stem cells.

To evaluate whether the radiotherapy- or chemotherapy-induced DNA repair could be an origin of CSCs, the X-ray, cisplatin or doxorubicin-treated GCNSCs were subjected to tumorsphere formation assay. The results indicated that the percentage of tumorsphere formation of X-ray, cisplatin or doxorubicin-treated GCNSCs was much higher than that of the control (GCNSCs without any treatment) (Fig. 2E). To confirm these results, a single cell from a tumorsphere of X-ray, cisplatin or doxorubicin-treated GCNSCs was subjected to tumorsphere formation assay. The results showed that the single cell could form a tumorsphere (Fig. 2F), indicating that the radiotherapy or chemotherapy treatment could be an origin of CSCs. To further confirm that the radiotherapy- or chemotherapy-treated GCNSCs could be an origin of GCSCs, the GCNSCs alone and the radiotherapy- or chemotherapy-treated GCNSCs were subjected to immunostaining analysis. The results demonstrated that the stemness markers Lgr5 and Klf4 were detected in the radiotherapy- or chemotherapy-treated GCNSCs but not in the control (GCNSCs alone) (Fig. 2G).

Taken together, the findings revealed that radiotherapy and chemotherapy caused DNA damage of cancer non-stem cells, thus promoting DNA repair and further leading to the biogenesis of cancer stem cells from cancer non-stem cells.

### Radiotherapy- or chemotherapy-induced DNA repair triggers the biogenesis of cancer stem cells of human solid tumors

**Fig. 3:**
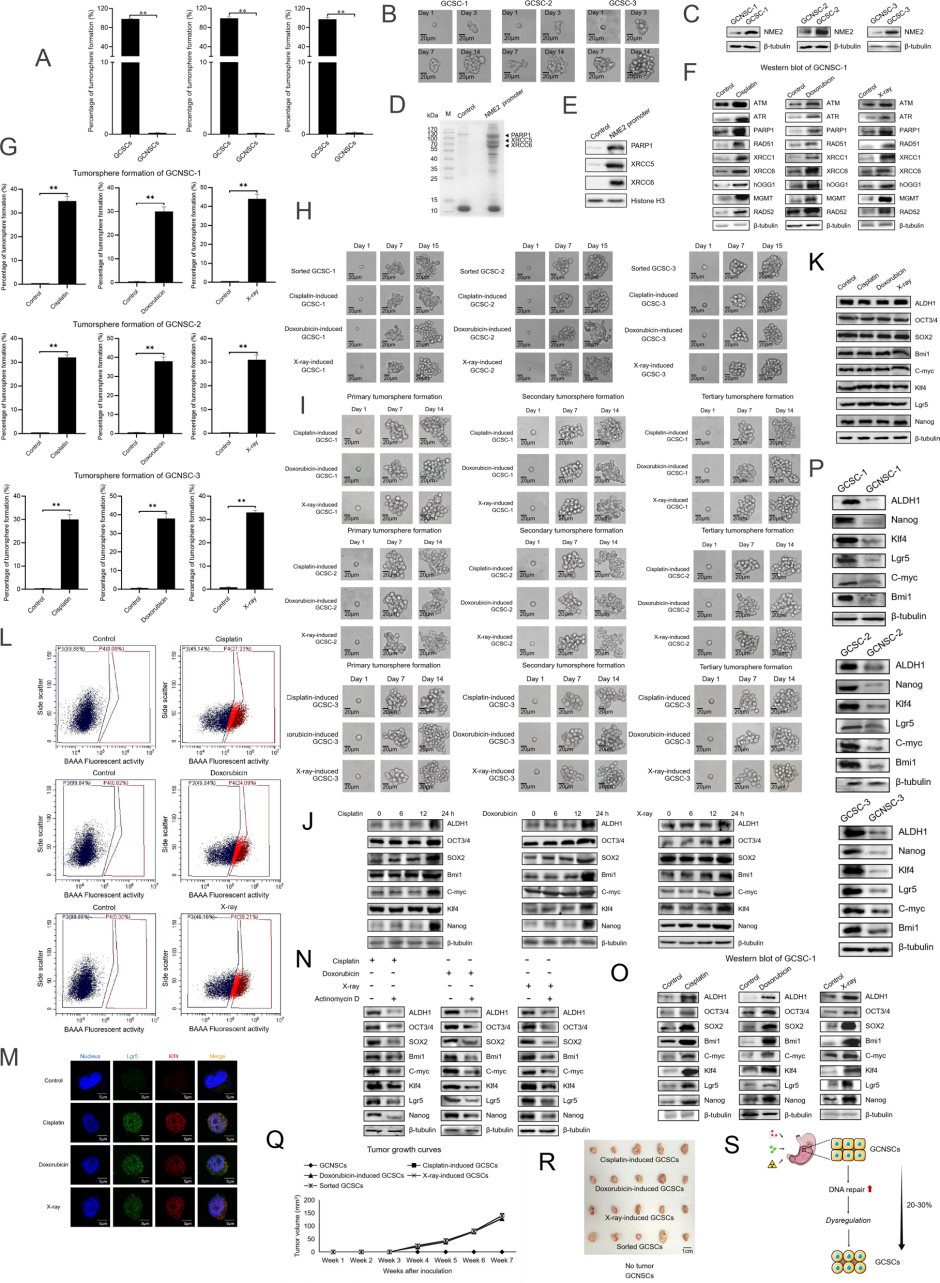
Radiotherapy- or chemotherapy-induced DNA repair triggers the biogenesis of cancer stem cells of human solid tumors. **A** Percentage of tumorsphere formation of gastric cancer stem cell-like cells (GCSCs) and non-stem cells (GCNSCs). Three GCSCs (GCSC-1, GCSC-2 and GCSC-3) and three GCNSCs (GCNSC-1, GCNSC-2 and GCNSC-3) were isolated from the solid tumors of three patients with gastric cancer, respectively. Then, the isolated potential GCSCs and GCNSCs were subjected to tumorsphere formation assays. **B** Tumorsphere formation assay of a single cell from GCSCs. Days indicated the culture time. **C** Upregulation of NME2 in gastric cancer stem-like cells (GCSCs). The expression level of NME2 in GCSCs and gastric cancer non-stem cells (GCNSCs) sorted from 3 gastric cancer patients was examined using Western blot. β-tubulin was used as a control. **D** Identification of the proteins interacted with the promoter of *NME2* gene. Nuclear proteins extracted from GCSC-1 were incubated with the biotin-labeled dsDNA of NME2 promoter and streptavidin-agarose beads. An intragenic double-stranded DNA coupled to streptavidin-agarose beads alone was used as a control. The proteins bound to the NME2 promoter were separated by SDS-PAGE with Coomassie staining and then identified by mass spectrometry. Arrows indicated the identified proteins bound to the NME2 promoter. M, protein marker. **E** Western blot of the proteins bound to the *NME2* promoter in GCSC-1. The proteins of the pulldown eluates using the *NME2* promoter were analyzed using Western blot. Histone H3 was used as a control. **F** Expressions of DNA repair genes in radiotherapy- or chemotherapy-treated gastric cancer non-stem cells. GCNSCs were treated with doxorubicin, cisplatin or X-ray. At 48 h after treatment, the gene expression in cells was examined with Western blot. The cells without any treatment were used as controls. β-tubulin was included in Western blots as a loading control. **G** Percentage of tumorsphere formation of radiotherapy- or chemotherapy-treated GCNSCs. The cells were treated with cisplatin, doxorubicin or X-ray and then cultured for 7 days. The tumorsphere formation efficiency of radiotherapy- or chemotherapy-treated GCNSCs was evaluated. The data were the average values of three independent experiments (*p < 0.01). **H** Tumorsphere formation of a single tumor cell. A single cell from a tumorsphere of radiotherapy- or chemotherapy-treated GCNSCs was subjected to tumorsphere formation assay. At different time after culture, the tumorsphere was examined. Scale bar, 20 μm. **I** Serial tumorsphere formation of a single cell from cisplatin-, doxorubicin- or X-ray-induced GCSCs. A single cell from cisplatin-, doxorubicin- or X-ray-induced GCSCs was subjected to primary tumorsphere formation assay. A single cell from a tumorsphere of primary tumorsphere formation assay was assayed in the secondary tumorsphere formation assay. A single cell from a tumorsphere of secondary tumorsphere formation assay was characterized in the tertiary tumorsphere formation assay. At different time after culture, the tumorsphere was examined. Scale bar, 20 μm. **J** Western blot analysis of stemness genes in the cisplatin-, doxorubicin- or X-ray-treated GCNSCs. GCNSCs were treated with cisplatin (300 μM), doxorubicin (0.5 μM) or X-ray (20 Gy). At different time after treatment, the expression levels of stemness genes in GCNSCs were detected using Western blot. β-tubulin served as a control. **K** Influence of DNA damage on the stemness of GCSCs. GCSCs were treated with cisplatin, doxorubicin and X-ray for 24 h. Then, the expression levels of stemness markers in GCSCs were determined using Western blot. β-tubulin was used as a control. **L** Flow cytometry plots showing ALDH activity of GCNSCs with or without cisplatin, doxorubicin or X-ray treatment. GCNSCs were treated with cisplatin, doxorubicin or X-ray for 48 h. The ALDH1-positive cells were GCSCs (P4 region) and the ALDH1-negative cells were GCNSCs (P3 region). **M** Representative images of immunofluorescence staining of GCNSCs treated with or without cisplatin, doxorubicin and X-ray. GCNSCs were treated with cisplatin, doxorubicin or X-ray for 48 h, and then the cells were analyzed using immunofluorescence analysis with the specific antibody against Lgr5 or Klf4. Nuclei were stained with DAPI. Scale bar, 5 μm. **N** Expression levels of stemness genes in radiotherapy- or chemotherapy-treated and actinomycin D-treated GCNSCs. GCNSCs were simultaneously treated with cisplatin, doxorubicin or X-ray and DNA repair inhibitor actinomycin D for 24 h. GCNSCs treated with cisplatin, doxorubicin or X-ray alone were used as controls. Then, the expression profiles of stemness genes in cells were examined using Western blot. **O** Examination of stemness genes’ expressions in radiotherapy- or chemotherapy-induced GCSCs using Western blot. β-tubulin was used as a control. **P** Upregulation of stemness genes in the sorted GCSCs. Western blot was conducted to examine the expressions of stemness genes in GCSCs and GCNSCs. **Q** Tumor growth curves in mice inoculated with the cisplatin-, doxorubicin- or X-ray-induced GCSCs, as well as the sorted GCSCs and GCNSCs. The tumor volumes of 5 mice of a treatment were evaluated every week. **R** Tumor sizes of different treatments. The mice inoculated different cells were killed at 50 day after the inoculation of cells. Scale bar, 1 cm. **S** Model for the chemotherapy- and radiotherapy-induced transition of gastric cancer non-stem cells to stem cells

To evaluate whether radiotherapy or chemotherapy triggered the biogenesis of cancer stem cells in clinic, the solid tumors from three patients with gastric cancer were subjected to the sorting of cancer stem cells and non-stem cells, generating three GCSCs (GCSC-1, GCSC-2 and GCSC-3). The cells collected from the tumorspheres in the tumorsphere formation assay were considered as GCSCs, while the cells which could not form tumorspheres were regarded as gastric cancer non-stem cells (GCNSCs). The results showed that the percentage of tumorsphere formation of the isolated potential GCSCs reached 98.2% (GCSC-1), 99.1% (GCSC-2) or 97.6% (GCSC-3), while no tumorsphere was observed for GCNSCs (Fig. 3A). A single cell from a tumorsphere of potential GCSCs could form a tumorsphere (Fig. 3B), indicating that GCSCs were obtained.

The Western blot results showed that NME2 was upregulated in GCSCs (GCSC-1, GCSC-2 and GCSC-3) compared with GCNSCs (GCNSC-1, GCNSC-2 and GCNSC-3) (Fig. 3C). At the same time, XRCC5, XRCC6 and PARP1 were specifically bound with the promoter of *NME2* (Fig. 3D). The Western blot data confirmed the interaction between 3 proteins and the promoter of *NME2* (Fig. 3E). These results were consistent with those in the gastric cancer stem cells were sorted from gastric cancer cell line MKN-45 (Fig. 1D–F), indicating that DNA repair was associated with tumorigenesis in clinic.

To explore the influence of radiotherapy and chemotherapy on the biogenesis of cancer stem cells from cancer non-stem cells, the GCNSCs isolated from the solid tumors from three patients with gastric cancer were treated with doxorubicin, cisplatin or X-ray, followed by the detection of DNA repair gene expressions. The results indicated that the DNA repair marker genes were significantly upregulated in the doxorubicin, cisplatin or X-ray-treated GCNSCs (Fig. 3F), showing that radiotherapy or chemotherapy promoted the DNA repair of cancer non-stem cells. At the same time, the results demonstrated that the percentage of tumorsphere formation of X-ray, cisplatin or doxorubicin-treated GCNSCs was more than 30%, whereas the percentage of tumorsphere formation of the control (GCNSCs without any treatment) was 0 (Fig. 3G). The single cell from a tumorsphere of X-ray, cisplatin or doxorubicin-treated GCNSCs could form a tumorsphere (Fig. 3H), indicating that radiotherapy or chemotherapy induced the biogenesis of GCSCs from non-stem cells. The serial tumorsphere formation of a single cell from cisplatin-, doxorubicin- or X-ray-induced GCSCs confirmed that radiotherapy or chemotherapy could induce the biogenesis of GCSCs (Fig. 3I). To explore what time the radiotherapy- or chemotherapy-triggered DNA repair could initiate the stemness of cancer cells, the expression profiles of stemness genes in the cisplatin-, doxorubicin- or X-ray-treated GCNSCs were detected at different time after radiotherapy or chemotherapy treatment. Western blot results demonstrated that the stemness genes were significantly upregulated in the cisplatin-, doxorubicin- or X-ray-treated GCNSCs only 24 h after radiotherapy or chemotherapy treatment (Fig. 3J). These data further confirmed the biogenesis of GCSCs resulted from the radiotherapy- or chemotherapy-triggered DNA repair of GCNSCs.

To evaluate the impact of DNA damage mediated by radiotherapy or chemotherapy on the expressions of stemness genes in GCSCs, GCSCs were treated with doxorubicin, cisplatin or X-ray and then the expression levels of stemness genes were examined. Western blots revealed that radiotherapy or chemotherapy did not affect the expression profiles of stemness in GCSCs (Fig. 3K), indicating that DNA damage had no influence on the stemness of GCSCs. To further explore whether GCSCs originated from the radiotherapy- or chemotherapy-treated GCNSCs, the radiotherapy- or chemotherapy-treated GCNSCs were characterized with flow cytometry and immunofluorescence analyses. The results of flow cytometry showed that the proportion of ALDH-positive cells in the radiotherapy- or chemotherapy-treated GCNSCs was 20–30%, while the proportion of ALDH-positive cells in GCNSCs alone (control) was 0% (Fig. 3L). At the same time, the immunofluorescence staining data indicated that the stemness markers Lgr5 and Klf4 were detected in the radiotherapy- or chemotherapy-treated GCNSCs but not in the control (GCNSCs alone) (Fig. 3M). These findings revealed that radiotherapy or chemotherapy promoted DNA repair, thus triggering the biogenesis of GCSCs from non-stem cells.

To evaluate whether the mutation mediated by DNA damage in radiotherapy- or chemotherapy-treated GCNSCs was involved in the biogenesis of GCSCs, the cisplatin-, doxorubicin- or X-ray-treated GCNSCs were treated with actinomycin D to inhibit DNA repair activity of GCNSCs, followed by the examination of stemness markers. Western blots indicated that the protein levels of stemness genes were significantly decreased in actinomycin D-treated cells compared with the controls (Fig. 3N). These data showed that DNA repair, but not mutation, was responsible for the biogenesis of GCSCs from the radiotherapy- or chemotherapy-treated GCNSCs.

To compare the sorted GCSCs, which were sorted from the solid tumors of three patients with gastric cancer, and the radiotherapy- or chemotherapy-induced GCSCs, which were induced from the GCNSCs isolated from the gastric cancer patients by doxorubicin, cisplatin or X-ray, the expression levels of stemness genes, the capacity of tumorsphere formation and tumorigenesis in mice of the two types of stem cells were characterized. The results indicated that the sorted GCSCs and the induced GCSCs (cisplatin-induced GCSCs, doxorubicin-induced GCSCs and X-ray-induced GCSCs) had a strong capacity of tumorsphere formation with a percentage close to 100% and the two types of stem cells were comparable (Fig. 3A, H). Western blots indicated that the stemness genes were significantly upregulated in the doxorubicin, cisplatin or X-ray-induced GCSCs (Fig. 3O), as well as in the sorted GCSCs (Fig. 3P), showing that the radiotherapy- or chemotherapy-induced GCSCs shared the same characteristics as the induced stem cells.

To evaluate the tumorigenic capacities of cisplatin-, doxorubicin- and X-ray-induced GCSCs and the sorted GCSCs, these cells were injected into nude mice, followed by tumor examination weekly. As shown in Fig. 3Q, the tumor growth curves of cisplatin-, doxorubicin- and X-ray-induced GCSCs in mice were similar to that of the sorted GCSCs. At the same time, the tumor sizes of cisplatin-, doxorubicin- and X-ray-induced GCSCs were comparable to that of the sorted GCSCs (Fig. 3R). However, there was no tumor observed in mice inoculated with non-stem cells (Fig. 3Q, R). These data indicated that the radiotherapy- or chemotherapy-induced GCSCs shared the same tumorigenic capacity as the sorted GCSCs.

The collective data demonstrated that the radiotherapy or chemotherapy treatment could induce the DNA repair of cancer non-stem cells of tumors to trigger dysregulation of cancer non-stem cells, leading to the biogenesis of GCSCs (Fig. 3S). About 20–30% GCNSCs of gastric solid tumors were reprogrammed into GCSCs induced by radiotherapy or chemotherapy (Fig. 3S).

### Underlying mechanism of radiotherapy- or chemotherapy-triggered biogenesis of cancer stem cells of solid tumors

**Fig. 4:**
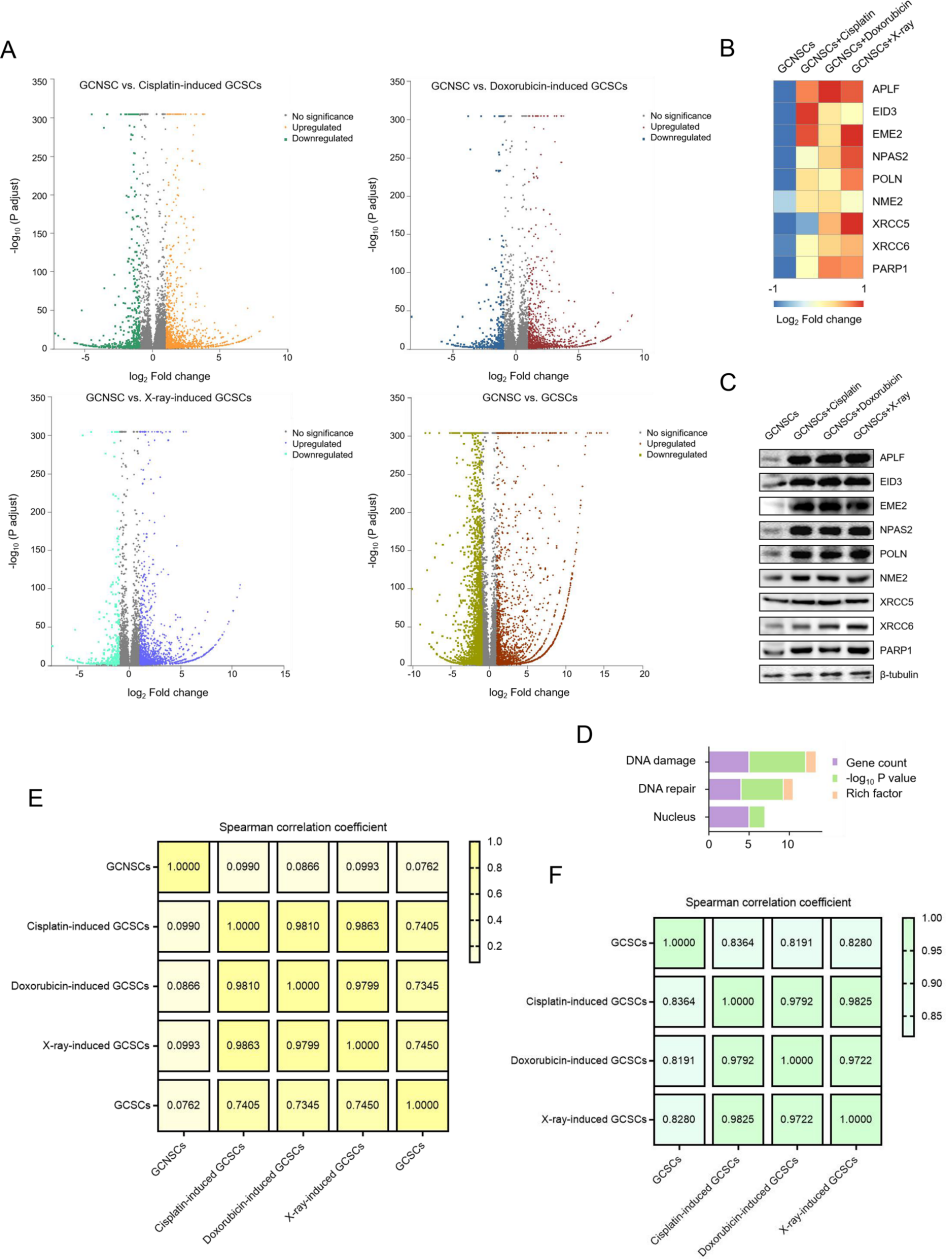
Underlying mechanism of radiotherapy- or chemotherapy-triggered biogenesis of cancer stem cells of solid tumors. **A** Volcano plots of differentially expressed genes between GCNSCs and cisplatin-induced GCSCs, doxorubicin-induced GCSCs, X-ray-induced GCSCs or the sorted GCSCs (*p* < 0.05). **B** Upregulations of APLF, EID3, EME2, NPAS2, POLN, XRCC5, XRCC6, PARP1 and NME2 genes in all the radiotherapy- and chemotherapy-treated GCNSCs. **C** Western blot analysis of APLF, EID3, EME2, NPAS2, POLN, XRCC5, XRCC6, PARP1 and NME2 proteins in GCNSCs with or without radiotherapy and chemotherapy (cisplatin, doxorubicin and X-ray treatments). β-tubulin was used as a control. **D** The biological function annotation of APLF, EID3, EME2, NPAS2 and POLN via the Universal Protein Resource (https://www.uniprot.org/) database. **E** Correlation analysis of stem-associated gene expression patterns between GCSCs, GCNSCs and cisplatin-induced, doxorubicin-induced or X-ray-induced GCSCs by Spearman correlation test. The strong positive correlation was reflected by Spearman correlation coefficient ranging from 0.6 to 1.0. **F** Correlation analysis of DNA repair gene expression patterns in the sorted GCSCs, cisplatin-induced GCSCs, doxorubicin-induced GCSCs and X-ray-induced GCSCs by Spearman correlation test

To reveal the mechanism of radiotherapy- or chemotherapy-triggered biogenesis of cancer stem cells of solid tumors, the transcriptome analyses of the sorted GCSCs from the solid tumors of a gastric patient and the doxorubicin, cisplatin or X-ray-induced GCSCs from the solid tumors, as well as GCNSCs of the solid tumors, were conducted. The results showed that 1926, 1642, 2067 and 4621 genes were differentially expressed between GCNSCs and cisplatin-induced GCSCs, doxorubicin-induced GCSCs, X-ray-induced GCSCs and the sorted GCSCs, respectively (Fig. 4A). Compared with GCNSCs, 11, 16 and 11 DNA repair genes were upregulated in cisplatin-induced, doxorubicin-induced and X-ray-induced GCSCs, respectively (Additional file 1: Fig. S1). Among them, APLF (aprataxin and PNKP like factor), EID3 (EP300 interacting inhibitor of differentiation 3), EME2 (essential meiotic structure-specific endonuclease subunit 2), NPAS2 (neuronal PAS domain protein 2) and POLN (DNA polymerase nu), NME2, XRCC5, XRCC6 and PARP1 genes were upregulated in all the radiotherapy- and chemotherapy-treated GCNSCs (Fig. 4B). Western blot analysis confirmed the upregulation of APLF, EID3, EME2, NPAS2, POLN, NME2, XRCC5, XRCC6 and PARP1 proteins in the radiotherapy- and chemotherapy-treated GCNSCs (Fig. 4C). However, APLF, EID3, EME2, NPAS2 and POLN were more significantly upregulated in the radiotherapy- and chemotherapy-treated GCNSCs than XRCC5, XRCC6 and PARP1 (Fig. 4C). The biological function annotation of APLF, EID3, EME2, NPAS2 and POLN using the Universal Protein Resource (https://www.uniprot.org/) database showed that the 5 proteins were remarkably enriched in DNA damage and DNA repair (Fig. 4D). These data indicated that APLF, EID3, EME2, NPAS2 and POLN played important roles in the radiotherapy- or chemotherapy-induced DNA repair of GCNSCs.

To explore whether the radiotherapy- or chemotherapy-induced GCSCs were comparable to the sorted GCSCs, the expression profiles of stemness genes of GCSCs were characterized and correlation analysis was conducted. The results showed that there was a strongly positive correlation between the sorted GCSCs and three types of induced GCSCs, whereas GCNSCs were not correlated with the sorted- or induced GCSCs (Fig. 4E). In addition to evaluation of stemness, the expression profiles of DNA repair genes within cisplatin-induced, doxorubicin-induced and X-ray-induced GCSCs were also positively correlated with that of the sorted GCSCs (Fig. 4F), hinting that DNA repair systems might play a role in cancer stem cells.

Taken together, these findings revealed that the radiotherapy or chemotherapy promoted the expression of DNA repair genes (APLF, EID3, EME2, NPAS2 and POLN) of gastric cancer non-stem stem cells to trigger DNA repair, resulting in the biogenesis of GCSCs.

### Role of EID3 in radiotherapy- or chemotherapy-triggered biogenesis of cancer stem cells

**Fig. 5:**
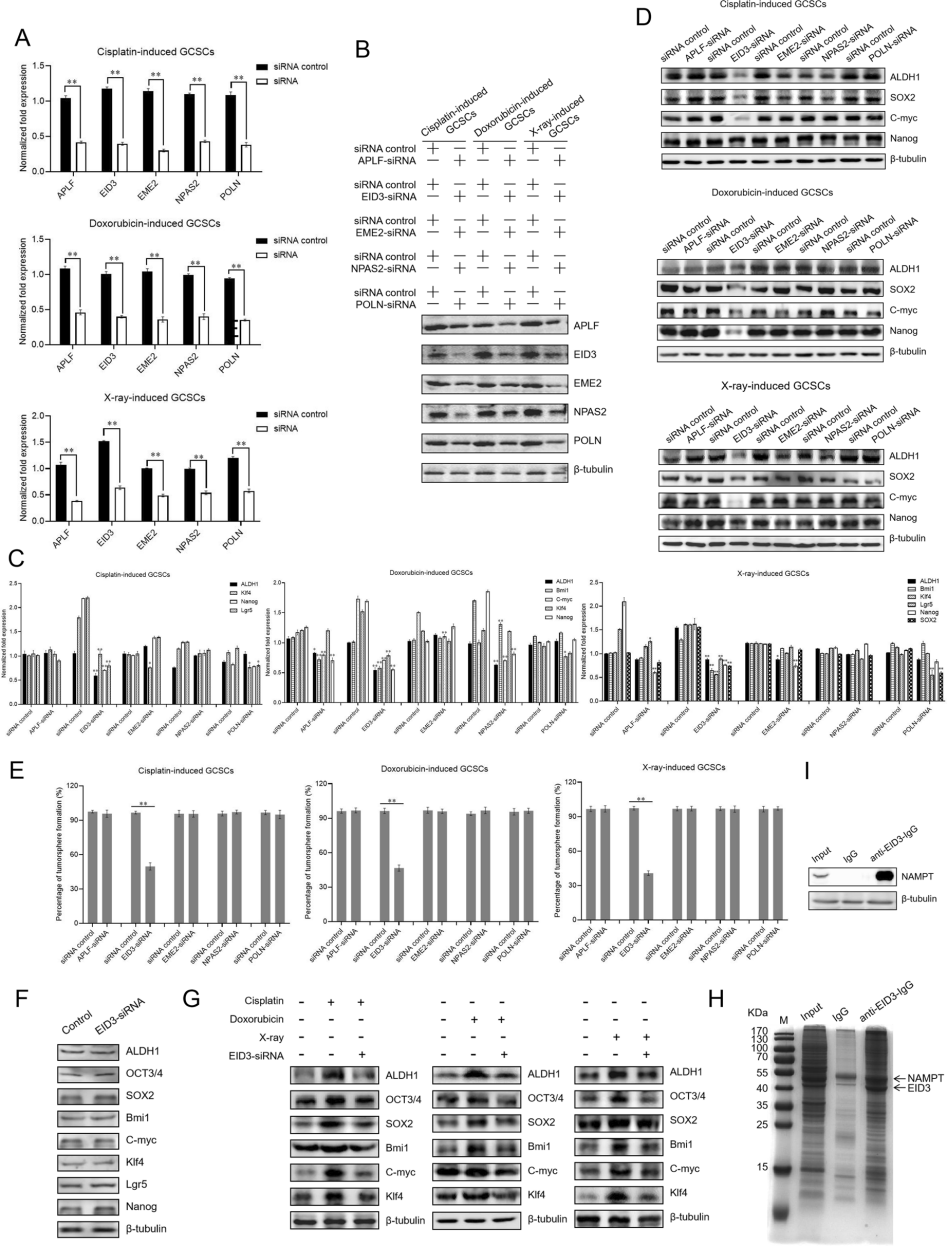

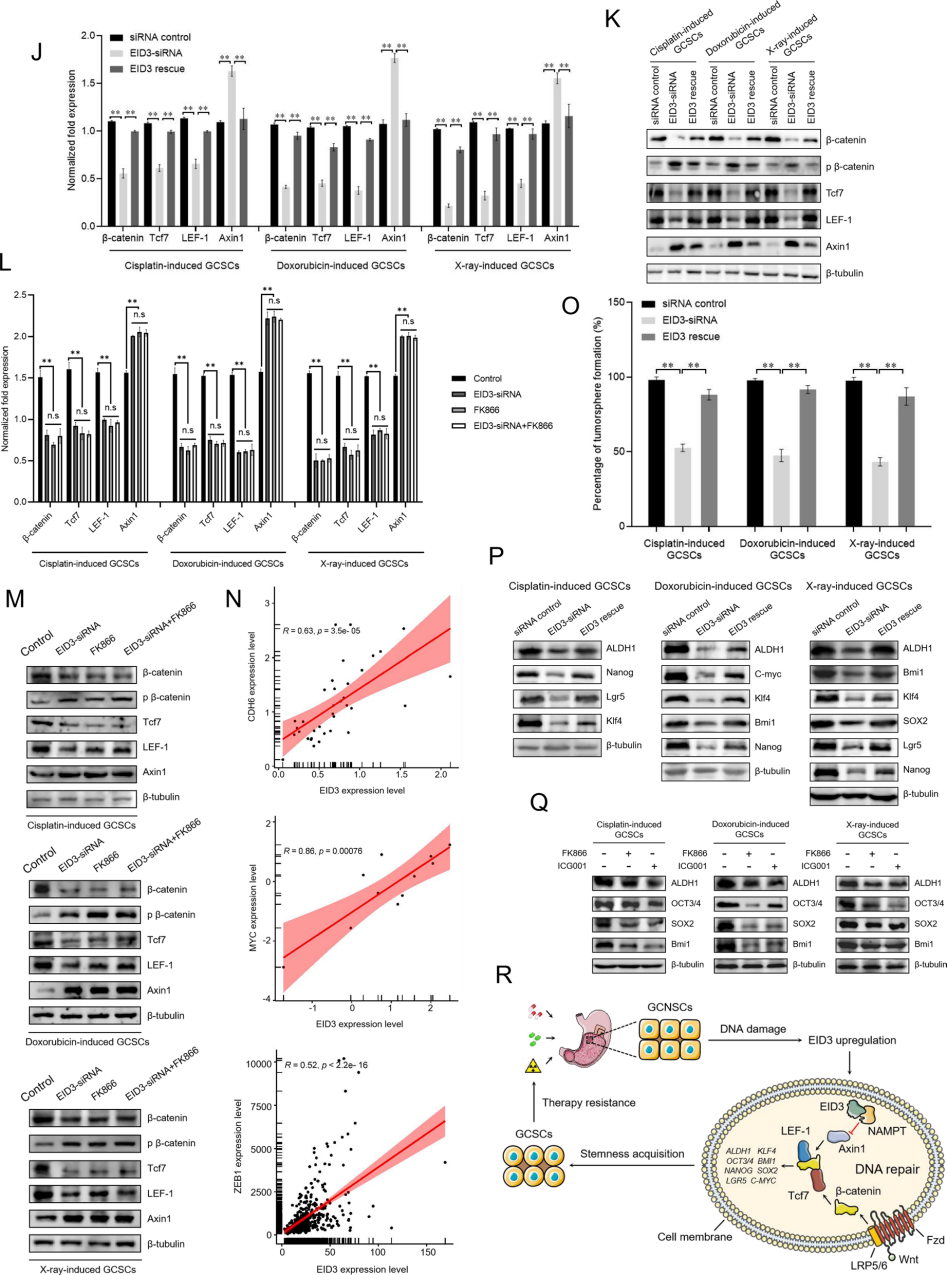
Role of EID3 in radiotherapy- or chemotherapy-triggered biogenesis of cancer stem cells. **A** and **B** Silencing of APLF, EID3, EME2, NPAS2 and POLN in cisplatin-induced, doxorubicin-induced or X-ray-induced GCSCs. GCNSCs were treated with cisplatin, doxorubicin or X-ray and transfected with gene-specific siRNA. As a control, siRNA control was included in the transfection. Thirty-six hours after transfection, the gene expression levels in GCSCs were evaluated using quantitative real-time PCR (**p < 0.01) (**A**) and Western blot (**B**). **C** and **D** Impact of gene silencing on the expression of stemness genes in cisplatin-induced, doxorubicin-induced or X-ray-induced GCSCs. The gene expression levels were examined 36 h after siRNA transfection with quantitative real-time PCR (**p < 0.01) (**C**) and Western blot (**D**). **E** Influence of gene knockdown on the tumorsphere formation capacity of cisplatin-induced, doxorubicin-induced and X-ray-induced GCSCs. GCSCs were transfected with gene-specific siRNA. As a control, siRNA control was included in the transfection. At the 7th day, the tumorsphere formation capability was examined. **F** Impact of EID3 silencing on the expressions of stemness genes in GCNSCs without DNA damage. EID3-siRNA was transfected into GCNSCs without DNA damage. Thirty-six hours later, the expression levels of stemness genes in GCNSCs were examined with Western blot. β-tubulin was used as a control. **G** Effects of EID depletion on the expressions of stemness genes in radiotherapy- or chemotherapy-treated GCNSCs. EID3 was knocked down in cisplatin-, doxorubicin- or X-ray-treated GCNSCs, followed by the examination of stemness genes’ expressions using Western blot. β-tubulin served as a control. **H** The protein bound to EID3. Coimmunoprecipitation (Co-IP) assay was conducted using EID3-specific antibody. The mouse IgG was used as a control. The Co-IP products were examined by SDS-PAGE with Coomassie staining. The proteins identified by mass spectrometry were indicated with arrows. M, protein marker. **I** Western blot analysis of the Co-IP products using EID3-specific antibody or the mouse IgG. β-tubulin was used as a control. **J** Effects of EID3 silencing and rescue on the NAMPT-mediated Wnt signaling pathway. The expression levels of the key components of Wnt pathway in EID3-silenced or rescued cisplatin-induced, doxorubicin-induced and X-ray-induced GCSCs were examined by quantitative real-time PCR. As a control, siRNA control was included in the experiments. The statistical significance of difference between treatments was indicated by asterisks (**p < 0.01). **K** Western blot analysis of the impact of EID3 silencing and rescue on the NAMPT-mediated Wnt signaling pathway. **L** Impact of EID3 silencing and/or NAMPT inhibition on the Wnt signaling pathway. In cisplatin-induced, doxorubicin-induced or X-ray-induced GCSCs, the expression of EID3 was knocked down and/or the NAMPT activity was inhibited by a specific inhibitor FK866. Then, the expression levels of the key components of Wnt pathway were evaluated by quantitative real-time PCR (**p < 0.01). **M** Western blot analysis of the impact of EID3 silencing and/or NAMPT inhibition on the Wnt pathway in cisplatin-induced, doxorubicin-induced or X-ray-induced GCSC. β-tubulin was used as a control. (**N**) Correlation analysis between EID3 and the Wnt pathways. The correlation analysis was carried out based on the cBioportal datasets of gastric cancers (https://www.cbioportal.org/). **O** Impact of EID3 silencing and rescue on the tumorsphere formation capacity of the radiotherapy- or chemotherapy-induced GCSCs. The cisplatin-induced, doxorubicin-induced or X-ray-induced GCSCs were transfected with EID3-siRNA and/or the plasmid expressing EID3 (EID3 rescue). At the 7th day after transfection, the percentage of tumorsphere formation of cells was evaluated (**p < 0.01). **P** Effects of EID3 silencing or rescue on the expressions of stemness genes in cisplatin-induced, doxorubicin-induced and X-ray-induced GCSCs. GCSCs were transfected with EID3-siRNA and/or the plasmid expressing EID3. Thirty-six hours after transfection, the expression levels of stemness genes in cells were examined using Western blot. β-tubulin was used as a control. **Q** Role of the Wnt signaling pathway in the EID3-mediated stemness of radiotherapy- or chemotherapy-induced GCSCs. The cisplatin-induced, doxorubicin-induced and X-ray-induced GCSCs were treated with FK866 (50 nM) or ICG001 (10 μM) for 24 h. Subsequently, the cells were subjected to Western blot analysis. β-tubulin served as a control. **R** Model for the radiotherapy- or chemotherapy-triggered biogenesis of cancer stem cells from cancer non-stem cells

To evaluate the roles of APLF, EID3, EME2, NPAS2 and POLN in radiotherapy- or chemotherapy-triggered biogenesis of GCSCs, the expression of these genes was, respectively, knocked down by gene-specific siRNA in radiotherapy- or chemotherapy-induced GCSCs, followed by the examination of the cell stemness. The results showed that the expressions of APLF, EID3, EME2, NPAS2 and POLN were knocked down in cisplatin-induced, doxorubicin-induced or X-ray-induced GCSCs (Fig. 5A, B). Among APLF, EID3, EME2, NPAS2 and POLN, only the silencing of EID3 led to the significant downregulations of all stemness genes examined in cisplatin-induced, doxorubicin-induced and X-ray-induced GCSCs compared with the controls (Fig. 5C, D), suggesting that EID3 played important roles in the radiotherapy- or chemotherapy-triggered biogenesis of GCSCs. The results of tumorsphere formation assays indicated that only the EID3 silencing significantly suppressed the tumorsphere forming ability of cisplatin-induced, doxorubicin-induced and X-ray-induced GCSCs (Fig. 5E). To explore the influence of EID3 depletion on the expressions of stemness genes in GCNCSs without DNA damage, EID3 was knocked down in GCNCSs and then the expression profiles of stemness genes were examined using Western blots. The results showed that the EID3 silencing had no effect on the expression levels of stemness genes in GCNCSs without DNA damage (Fig. 5F). To assess the influence of EID3 silencing on the stemness of radiotherapy- or chemotherapy-treated GCNSCs, EID3 was knocked down in cisplatin-, doxorubicin- or X-ray-treated GCNSCs, followed by the examination of stemness genes’ expressions. Western blots demonstrated that radiotherapy or chemotherapy promoted the expressions of stemness genes in GCNSCs, while the EID3 silencing led to the suppression of stemness genes’ expressions in radiotherapy- or chemotherapy-treated GCNSCs (Fig. 5G), indicating that the influence of radiotherapy- or chemotherapy-triggered DNA repair on the stemness of radiotherapy- or chemotherapy-treated GCNSCs was mediated by EID3. In this context, EID3 was further characterized.

To reveal the role of EID3 in the radiotherapy- or chemotherapy-triggered biogenesis of GCSCs, the proteins interacted with EID3 were characterized. The coimmunoprecipitation (Co-IP) data indicated that a specific protein was obtained when EID3-specific antibody was used (Fig. 5H). Based on mass spectrometry analysis, the protein was identified to be nicotinamide phosphoribosyltransferase (NAMPT) (Fig. 5H). Western blot analysis confirmed the mass spectrometric data (Fig. 5I). As reported, NAMPT can promote the Wnt/β-catenin signaling pathway [26]. To reveal whether EID3 was involved in the Wnt signaling pathway, the expression of EID3 was silenced or rescued in cisplatin-induced, doxorubicin-induced and X-ray-induced GCSCs, followed by the examination of the Wnt signaling pathway. The results indicated that the EID3 knockdown led to significant decreases in β-catenin, Tcf7 (transcription factor 7) and LEF-1 (lymphoid enhancer-binding factor 1) and a significant increase in Axin1, key proteins of Wnt pathway, in cisplatin-induced, doxorubicin-induced and X-ray-induced GCSCs compared with the control (siRNA control) (Fig. 5J, K). At the same time, the expressions of β-catenin, Tcf7 and LEF-1 were recovered and the expression of Axin1 was inhibited in cells by EDI3 rescue (Fig. 5J, K). The EID3 silencing promoted the phosphorylation of β-catenin (Fig. 5K). These data demonstrated that EID3 was associated with the NAMPT-mediated Wnt/β-catenin signaling pathway.

To investigate whether the involvement of EID3 in the Wnt signaling pathway was related to the interaction between EID3 and NAMPT, the EID3 expression was silenced and/or the NAMPT activity was suppressed in cisplatin-induced, doxorubicin-induced or X-ray-induced GCSCs, followed by the examination of the Wnt pathway. The results of quantitative real-time PCR revealed that the NAMPT inhibition markedly suppressed the Wnt signaling pathway (Fig. 5L). Western blots essentially yielded the similar results (Fig. 5M). These data indicated that the involvement of EID3 in the Wnt pathway was related to its interaction with NAMPT.

To further explore the relationship between EID3 and the Wnt pathway, the correlation analysis was performed based on the cBioportal datasets of gastric cancers (https://www.cbioportal.org/). The results demonstrated that the EID3 expression level was positively correlated with the expression levels of CDH6, MYC and ZEB1, the crucial molecules of Wnt pathway (Fig. 5N), showing that EID3 was involved in the activation of the Wnt pathway.

It is reported that the Wnt signaling pathway facilitates the reprogramming of cancer cells to promote stemness and is involved in the maintenance of stemness of cancer stem cells [27]. Therefore, the influence of EID3 on the stemness acquisition of the radiotherapy- or chemotherapy-induced GCSCs was characterized. The data of tumorsphere formation assays indicated that the EID3 silencing significantly suppressed the tumorsphere formation capacity, and the tumorsphere formation capacity of the treatment EID3 rescue was comparable to that of the control (Fig. 5O). At the same time, the results indicated that the EID3 silencing led to significant downregulation of stemness genes in cisplatin-induced, doxorubicin-induced and X-ray-induced GCSCs, while the EID3 rescue recovered the expressions of stemness genes in the radiotherapy- and chemotherapy-induced GCSCs (Fig. 5P). These data revealed that EID3 played a positive role in the stemness acquisition of the radiotherapy- or chemotherapy-induced GCSCs. To evaluate whether the Wnt signaling pathway was involved in the EID3-mediated stemness of GCSCs, the cisplatin-induced, doxorubicin-induced and X-ray-induced GCSCs were treated with FK866 or ICG001, the inhibitor of NAMPT or β-catenin, followed by the examination of stemness genes. The results showed that the treatment of FK866 or ICG001 led to downregulation of stemness genes in cisplatin-induced, doxorubicin-induced and X-ray-induced GCSCs (Fig. 5Q), indicating that the Wnt signaling pathway was responsible for the EID3-mediated stemness of GCSCs.

Collectively, these findings indicated that the radiotherapy- or chemotherapy-induced DNA damage of GCNSCs upregulated the EID3 expression and the interaction between EID3 and NAMPT activated the Wnt signaling pathway to promote the expressions of stemness genes, thus triggering the biogenesis of GCSCs (Fig. 5R).

### Requirement of EID3 for the stemness of gastric cancer stem cell-like cells

**Fig. 6:**
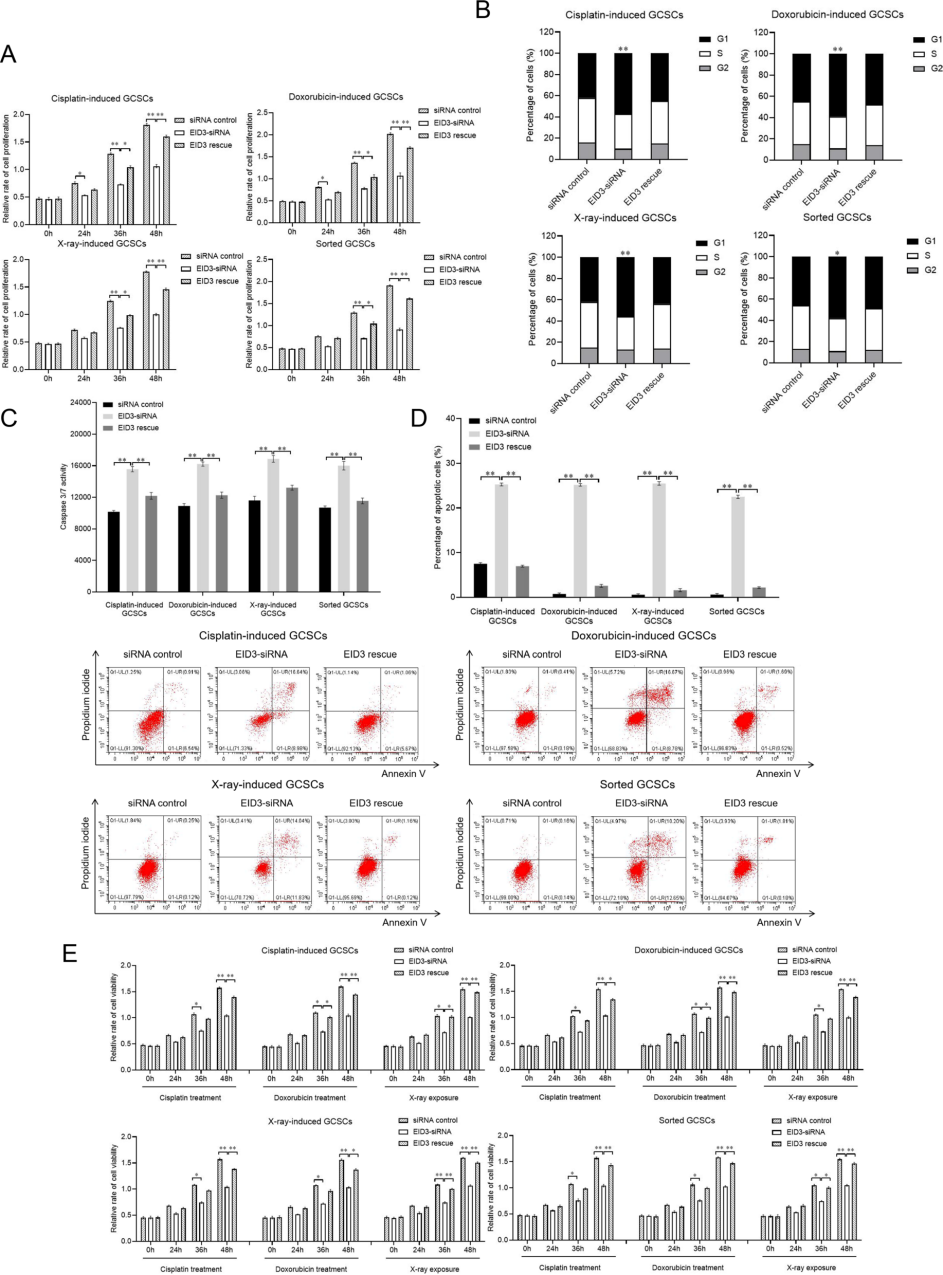
Requirement of EID3 for the stemness of gastric cancer stem cell-like cells. **A** Role of EID3 in the proliferation of radiotherapy-induced, chemotherapy-induced or sorted GCSCs. GCSCs were transfected with EID3-siRNA. As a control, siRNA control was included in the transfection. At different time after transfection, the cell proliferation was examined. The statistical significance of difference between treatments was indicated with asterisks (*p < 0.05; **p < 0.01). **B** Effects of EID3 silencing or rescue on cell cycle of radiotherapy-induced, chemotherapy-induced or sorted GCSCs. Thirty-six hours after the treatment of EID3-siRNA or rescue, GCSCs were subjected to cell cycle analysis (*p < 0.05; **p < 0.01). **C** Impact of EID3 silencing or rescue on the caspase 3/7 activity of GCSCs. GCSCs were treated with EID3-siRNA or EID3 rescue. Thirty-six hours after treatment, the caspase 3/7 activity of cells was examined (**p < 0.01). **D** Evaluation of apoptosis using annexin V assays. Thirty-six hours after treatment, apoptosis of GCSCs was examined using flow cytometry (**p < 0.01). **E** Role of EID3 in the therapy resistance of gastric cancer stem cell-like cells (GCSCs). GCSCs (cisplatin-induced, doxorubicin-induced, X-ray-induced or sorted GCSCs) were transfected with EID3-siRNA, siRNA control or EID3-siRNA + plasmid expressing EID3. At different time after transfection (0, 24, 36 and 48 h), GCSCs were treated with cisplatin (300 μM, 24 h), doxorubicin (1 μM, 24 h) or X-ray (20 Gy). Subsequently, the cell viability was examined (*p < 0.05; **p < 0.01)

To explore the influence of EID3 on the stemness of GCSCs, EID3 was knocked down or rescued in the cisplatin-induced, doxorubicin-induced, X-ray-induced or sorted GCSCs, followed by the examinations of proliferation and therapy resistance of GCSCs. The results indicated that the EID3 silencing significantly suppressed the proliferation of cisplatin-induced, doxorubicin-induced, X-ray-induced or the isolated GCSCs compared with the controls, while the EID3 rescue recovered the GCSCs’ proliferation (Fig. 6A). These data demonstrated that EID3 played an essential role in the proliferation of radiotherapy-induced, chemotherapy-induced and sorted GCSCs. The flow cytometry data showed that the percentage of EID3-knocked-down radiotherapy-induced, chemotherapy-induced or sorted GCSCs in the G1 phase was significantly increased compared with the control (Fig. 6B), indicating that the EID3 silencing triggered cell cycle arrest in G1 phase of cisplatin-induced, doxorubicin-induced, X-ray-induced and sorted GCSCs. The cell cycle profile of EID3-rescued radiotherapy-induced, chemotherapy-induced or sorted GCSCs was comparable to that of the control (Fig. 6B). These results revealed that the proliferation suppression of radiotherapy-induced, chemotherapy-induced or sorted GCSCs by EID3 silencing results from the cell cycle arrest in G1 phase.

To unravel whether the cell cycle arrest of GCSCs induced apoptosis, the EID3-silenced or rescued cisplatin-induced, doxorubicin-induced, X-ray-induced or sorted GCSCs were subjected to apoptotic examination. The data of caspase 3/7 activity detection showed that the EID3 knockdown significantly increased the caspase 3/7 activity of GCSCs compared with the control, while the caspase 3/7 activity of EID3-rescued GCSCs was similar to that of the control (Fig. 6C). The annexin V assays yielded the similar results (Fig. 6D). These data indicated that the EID3 silencing promoted apoptosis of GCSCs.

To evaluate the role of EID3 in the therapy resistance, the expression of EID3 was knocked down or rescued in cisplatin-induced, doxorubicin-induced, X-ray-induced or sorted GCSCs, followed by the radiotherapy or chemotherapy treatment of GCSCs. The results revealed that the EID3 deficiency led to a significant decrease in the viability of the radiotherapy- or chemotherapy-treated GCSCs compared with the control, while the EID3 rescue recovered the viability of the radiotherapy- or chemotherapy-treated GCSCs (Fig. 6E). These data indicated that EID3 played a positive role in the therapy resistance of GCSCs.

Taken together, these findings indicated that EID3 was required for the maintenance of the stemness of the radiotherapy- or chemotherapy-induced GCSCs.

### Influence of EID3 on tumorigenesis of GCSCs in vivo

**Fig. 7:**
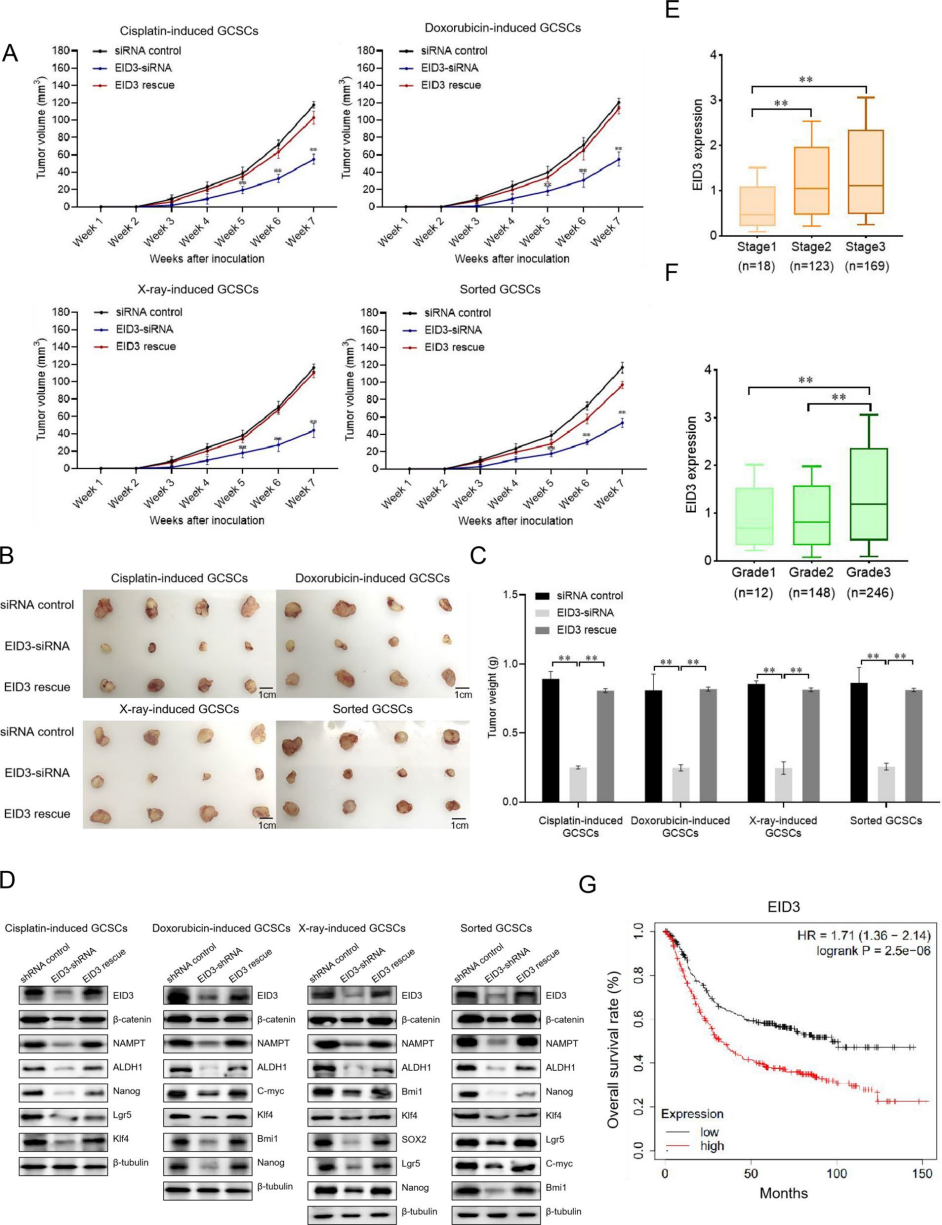
Influence of EID3 on tumorigenesis of GCSCs in vivo*.* **A** Role of EID3 in tumor growth in mice. GCSCs (cisplatin-induced, doxorubicin-induced, X-ray-induced or sorted GCSCs), transfected with EID3-siRNA, EID3 rescue or siRNA control, were injected into nude mice. The tumor volume in mice was measured every week for a 7-week period. **B** Tumor sizes of mice with different treatments. **C** Tumor weights of mice injected with GCSCs transfected with EID3-siRNA, EID3 rescue or siRNA control. **D** Protein levels of EID3, β-catenin, NAMPT and stemness signatures in the solid tumors of the mice injected with the EID3-silenced or the EID3-rescued GCSCs (cisplatin-induced, doxorubicin-induced, X-ray-induced or sorted GCSCs). The proteins were examined with Western blot. Β-tubulin was used as a control. **E** The relationship between the EID3 expression level and the histological grades of gastric cancer patients. The EID3 expression level and the pathological stages of gastric cancer patients were characterized using The Cancer Genome Atlas (TCGA). Stage 1, cancer limited to single cancer tissue (*n* = 18); Stage 2, partially infiltrating cancer (*n* = 123); Stage 3, cancer with lymphatic metastasis (*n* = 169). **F** The relationship between the EID3 expression level and the histological grades of gastric cancer patients. Grade 1, well differentiated (low grade); Grade 2, moderately differentiated (intermediate grade); Grade 3, poorly differentiated (high grade). **G** Kaplan–Meier analysis of gastric cancer patients with high and low expression level of EID3. EID3-low group, *n* = 282; EID3-high group, *n* = 349; HR, hazard ratio. In all panels, the statistical significance of differences between treatments was indicated with asterisks (*p < 0.05; **p < 0.01)

To evaluate the role of EID3 in tumorigenesis of GCSCs in vivo, the EID3-siRNA, EID3 rescue or siRNA control-transfected GCSCs were injected into nude mice, followed by the examination of tumor growth. The results indicated that the tumor growth in the mice injected with the EID3-silenced GCSCs was significantly repressed compared with that in the control mice, while the tumor growth in the mice treated with EID3 rescue was comparable to that of the control (Fig. 7A). The examination of tumor size and tumor weight generated the similar results (Fig. 7B, C). These data revealed that the EID3 silencing suppressed tumorigenesis of the radiotherapy- or chemotherapy-induced GCSCs in vivo.

Western blot data showed that the expression levels of EID3, NAMPT, β-catenin, and stemness signatures were remarkably downregulated in the solid tumors of the mice with EID3-shRNA compared with the control, while the treatment of EID3 rescue yielded the similar results to those of the control (Fig. 7D). These data demonstrated that EID3 promoted tumorigenesis of GCSCs in vivo via the EID3-NAMPT-Wnt/β-catenin axis.

To evaluate whether EID3 was associated with tumor development in clinic, the relationship between the EID3 expression level and the tumor progression or the overall survival of gastric cancer patients was characterized by mining online databases. The results showed that the higher the tumor histological grades and pathological stages of gastric cancer patients were, the higher the EID3 expression level was (Fig. 7E, F). Kaplan–Meier survival analysis revealed that the high level of EID3 was remarkably correlated with poor overall survival of gastric cancer patients (Fig. 7G). These data indicated that EID3 played a positive role in tumor development of gastric cancer in clinic.

Collectively, these findings revealed that EID3 promoted tumorigenesis of GCSCs in vivo via the EID3-NAMPT-Wnt/β-catenin axis.

## Discussion

It is well known that CSCs, a rare and relatively quiescent subpopulation of cancer cells which exhibit high tumorigenic potency and are endowed with self-renewal and differentiation capability, are responsible for initiating oncogenesis, tumor progression, and metastatic growth [28, 29]. CSCs are the major players for therapy resistance and cancer recurrence [28, 29]. In clinic, it is found that the relapse of cancer, after initially effective routine therapies, is fueled by the preexisting radio/chemo-resistant CSCs [30, 31]. These reports indicate that there is a relationship between the cancer relapse and CSCs. As reported, the resistance to radiation or chemotherapy as well as inevitable disease relapse driven by CSCs attributes to the upregulation of drug transporters, the improvement in DNA repair capacity and the adaption to reactive oxygen species (ROS) [32, 33]. Nevertheless, it remains unclear whether radiotherapy and chemotherapy contributed to the neoplasm recurrence via the de novo biogenesis of CSCs in cancer patients treated with radiotherapy and chemotherapy. In this study, the findings manifested that chemoradiotherapy could trigger faulty DNA repair process, which in turn induced the dedifferentiation of cancer non-stem cells, leading to the biogenesis of CSCs. Our study showed that chemoradiotherapy could make 20% to 27% tumor non-stem cells of solid tumors from gastric cancer patient to change into cancer stem cell-like cells, which was much higher than the proportion of cancer stem cell-like cells in solid tumors. As reported, there are merely 4.5% cancer stem cells in the solid tumors of gastric cancer patients [34]. In clinic, therefore, the surgical treatment must radically excise all the tumors and the periphery abnormal tissues of a patient. If the removal of tumors by surgical treatment is not radical, the chemoradiotherapy of a cancer patient can induce DNA damage of tumor cells, resulting in the aberration of DNA repair of differentiated tumor cells and thus contributing to the biogenesis of CSCs from cancer non-stem cells.

DNA double-strand breaks, caused by ionizing radiation and cytotoxic chemotherapy which are widely exploited for routine treatments within most cancers, trigger DNA repair of cancer cells [35]. Some reports find that DNA repair genes are upregulated in CSCs, leading to therapy resistance mediated by enhanced DNA repair machinery [36, 37, 38]. However, the underlying mechanism of DNA repair inducing the biogenesis of CSCs is still unclear. In this study, the findings indicated that EID3 bridged DNA repair and the biogenesis of CSCs. EID3, which was involved in DNA repair process [39], was upregulated in the cisplatin-induced, doxorubicin-induced and X-ray-induced GCSCs. The further data demonstrated that EID3 bound to the nicotinamide phosphoribosyltransferase (NAMPT), a novel modulator of Wnt pathway, leading to the activation of Wnt signaling pathway [26]. Our correlation analysis also revealed that EID3 was positively correlated with the Wnt pathway in gastric cancers, presenting a new role of EDI3 in the Wnt pathway. As reported, the activation of Wnt pathway triggers the generation of CSCs, including breast cancer, liver cancer, colorectal cancer and esophageal squamous cell carcinoma [40]. Our results showed that the activation of Wnt pathway promoted the expressions of stemness genes, thus contributing to the stemness acquisition of the radiotherapy- or chemotherapy-induced GCSCs. In this context, our findings revealed a novel underlying mechanism for the biogenesis of CSCs from the differentiated tumor cells.

## Conclusion

Our data indicated that EID3 was interacted with NAMPT to activate the Wnt signaling pathway which played a part in stemness acquisition, thus triggering the biogenesis of cancer stem cell-like cells, which might provide novel molecular insights into the biogenesis of cancer stem cell-like cells from the differentiated tumor cells.

## Supplementary Information

**Additional file 1. Fig. S1**: Heat map of the differentially expressed DNA repair genes between GCNSCs and cisplatin-induced, doxorubicin-induced or X-ray-induced GCSCs.

## Data Availability

The datasets used and/or analyzed during the current study are available from the corresponding author on reasonable request.

## Abbreviations

CSCs: Cancer stem cells
GCNSCs: Gastric cancer non-stem cells
GCSCs: Gastric cancer stem cell-like cells
EID3: EP300 interacting inhibitor of differentiation 3
NAMPT: Nicotinamide phosphoribosyltransferase
EMT: Epithelial–mesenchymal transition
KLF4: Kruppel-like factor 4
OCT3/4: Octamer-binding transcription factor 3/4
NME2: NME/NM23 nucleoside diphosphate kinase 2
FACS: Fluorescence-activated cell sorting
ATCC: American Type Culture Collection
ALDH1: Aldehyde dehydrogenase 1
BAAA: BODIPY-aminoacetate
DEAB: Diethylaminobenzaldehye
DMEM: Dulbecco’s modified eagle medium
FBS: Fetal bovine serum
DMEM/F12: Dulbecco’s modified eagle medium/nutrient mixture F12
EGF: Epidermal growth factor
bFGF: Basic fibroblast growth factor
Trypsin-EDTA: Trypsin–ethylenediaminetetraacetic acid
PBS: Phosphate-buffered saline
NP-40: Nonidet P-40
PVDF: Polyvinylidene difluoride
ERCC4: Excision repair 4, endonuclease catalytic subunit
XPD: Xeroderma pigmentosum D
BRCA1: Breast cancer susceptibility 1
BRCA2: Breast cancer susceptibility 2
PARP1: Poly ADP-ribose polymerase 1
RAD51: RAS associated with diabetes protein 51
SOX2: SRY-box 2
GAPDH: Glyceraldehyde-3-phosphate dehydrogenase
TCGA: The Cancer Genome Atlas
PMSF: Phenylmethylsulfonyl fluoride
Co-IP: Coimmunoprecipitation
CCK-8: Cell counting kit-8
APLF: Aprataxin and PNKP-like factor
EME2: Essential meiotic structure-specific endonuclease subunit 2
NPAS2: Neuronal PAS domain protein 2
POLN: DNA polymerase nu
KEGG: Kyoto Encyclopedia of Genes and Genomes
PI: Propidium iodide
FITC: Fluorescein isothiocyanate
SD: Standard deviations
ANOVA: Analysis of variance
