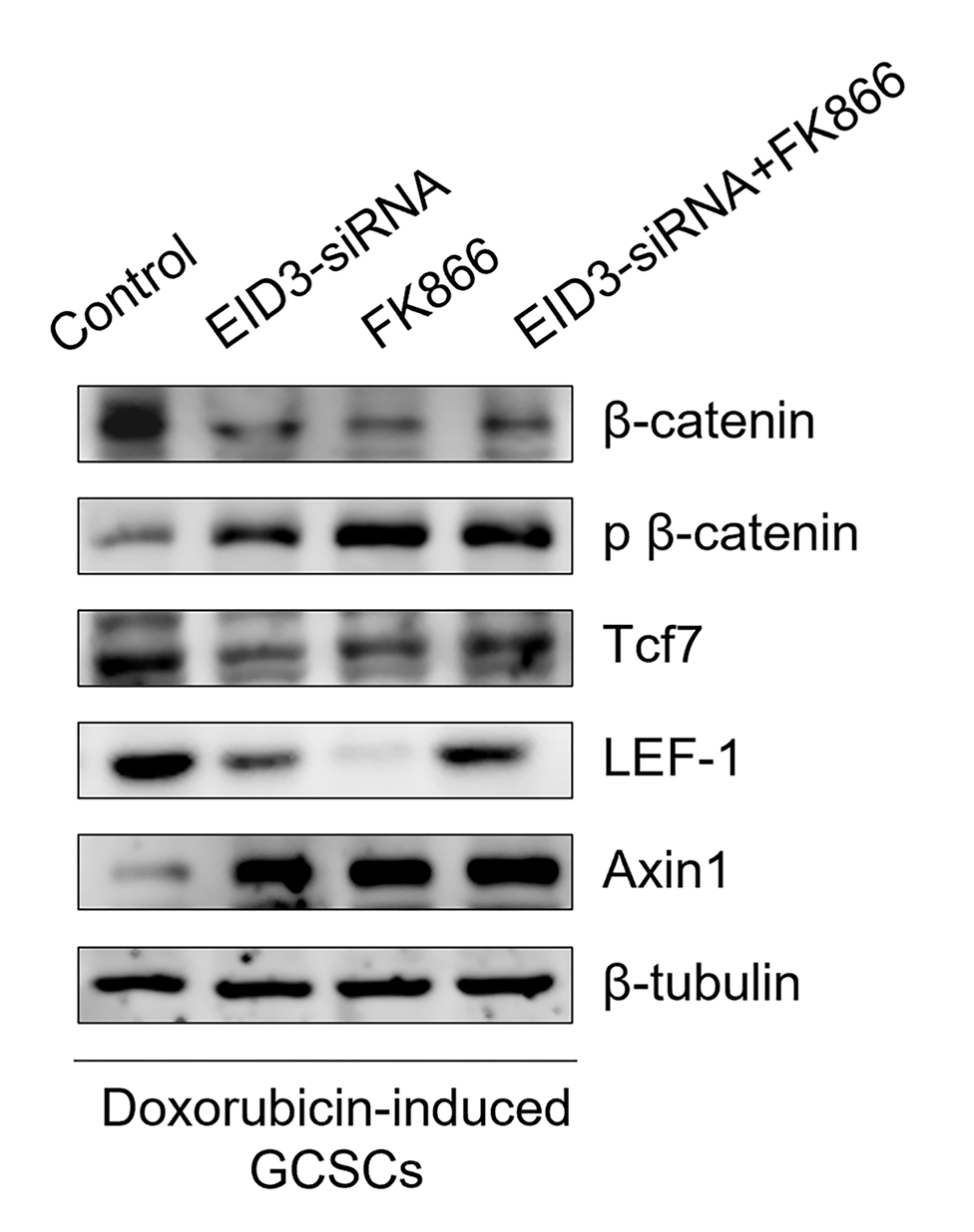# Correction: Radiochemotherapy-induced DNA repair promotes the biogenesis of gastric cancer stem cells

**DOI:** 10.1186/s13287-024-03984-x

**Published:** 2024-10-10

**Authors:** Yu Lu, Xiaobo Zhang

**Affiliations:** College of Life Sciences, Laboratory for Marine Biology and Biotechnology of Pilot National Laboratory for Marine Science and Technology (Qingdao) and Southern Marine Science and Engineering Guangdong Laboratory (Zhuhai), Zhejiang University, Hangzhou, 310058 People’s Republic of China

**Stem Cell Research & Therapy (2022) 13:481**

10.1186/s13287-022-03165-8

In Fig. 5M of the original article, the image of the fourth lane (LEF-1) of the second picture (Doxorubicin-induced GCSCs) was inadvertently replaced with an incorrect version during the upload process. The authors wish to note a correction to the aforementioned picture via the corrected picture ahead in this Correction article.

The authors deeply regret that this error occurred and sincerely apologize for any inconvenience.