# Supplementary material for: Radiochemotherapy-induced DNA repair promotes the biogenesis of gastric cancer stem cells
Source: Stem Cell Res Ther. 2022 Sep 24;13:481. doi: 10.1186/s13287-022-03165-8 (PMC9509583; doi:10.1186/s13287-022-03165-8)

Supplementary materials

Fig. S1. Heat-map of the differentially expressed DNA repair genes between GCNSCs and cisplatin-induced, doxorubicin-induced or X-ray-induced GCSCs.

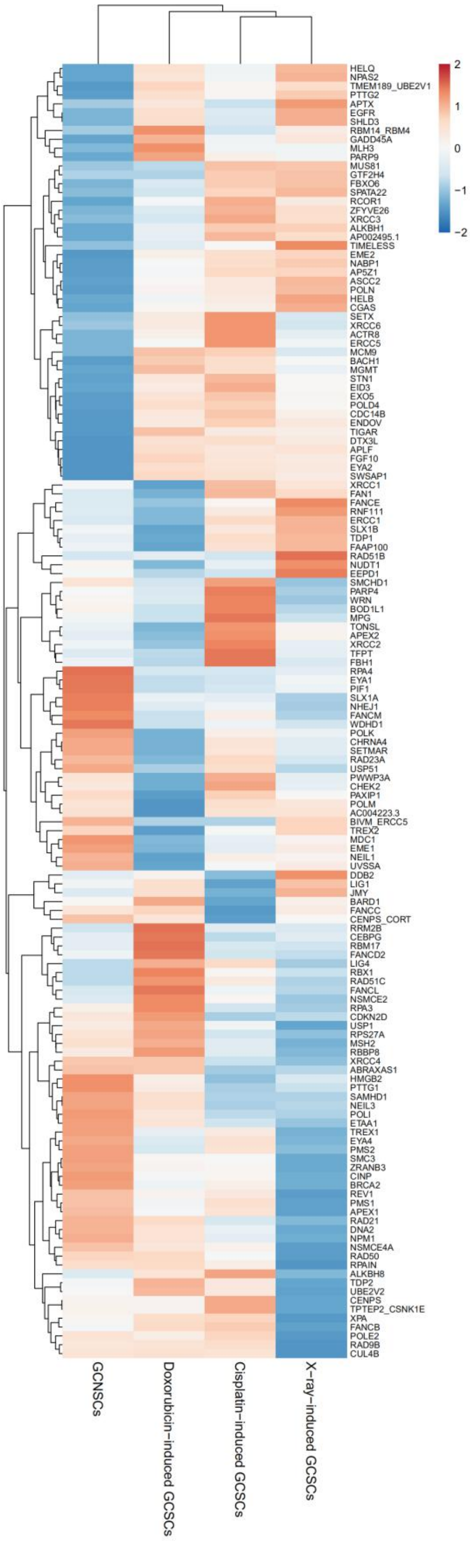

Supplement: Supplementary file 1 — Additional file 1. Fig. S1: Heat map of the differentially expressed DNA repair genes between GCNSCs and cisplatin-induced, doxorubicin-induced or X-ray-induced GCSCs. [file 13287_2022_3165_MOESM1_ESM.pdf]
